# QSPR models for predicting the adsorption capacity for microplastics of polyethylene, polypropylene and polystyrene

**DOI:** 10.1038/s41598-020-71390-3

**Published:** 2020-09-03

**Authors:** Miao Li, Haiying Yu, Yifei Wang, Jiagen Li, Guangcai Ma, Xiaoxuan Wei

**Affiliations:** College of Geography and Environmental Sciences, Zhejiang Normal University, Yingbin Avenue 688, Jinhua, 321004 China

**Keywords:** Environmental impact, Environmental chemistry

## Abstract

Microplastics have become an emerging concerned global environmental pollution problem. Their strong adsorption towards the coexisting organic pollutants can cause additional environmental risks. Therefore, the adsorption capacity and mechanisms are necessary information for the comprehensive environmental assessments of both microplastics and organic pollutants. To overcome the lack of adsorption information, five quantitative structure–property relationship (QSPR) models were developed for predicting the microplastic/water partition coefficients (log *K*_d_) of organics between polyethylene/seawater, polyethylene/freshwater, polyethylene/pure water, polypropylene/seawater, and polystyrene/seawater. All the QSPR models show good fitting ability (*R*^2^ = 0.811–0.939), predictive ability (*Q*^2^_ext_ = 0.835–0.910, *RMSE*_ext_ = 0.369–0.752), and robustness (*Q*_cv_^2^ = 0.882–0.957). They can be used to predict the *K*_d_ values of organic pollutants (such as polychlorinated biphenyls, chlorobenzene, polycyclic aromatic hydrocarbons, antibiotics perfluorinated compounds, etc.) under different pH conditions. The hydrophobic interaction has been indicated as an important mechanism for the adsorption of organic pollutants to microplastics. In sea waters, the role of hydrogen bond interaction in adsorption is considerable. For polystyrene, π–π interaction contributes to the partitioning. The developed models can be used to quickly estimate the adsorption capacity of organic pollutants on microplastics in different types of water, providing necessary information for ecological risk studies of microplastics.

## Introduction

Microplastics, defined as plastics with particle size < 5 mm, have become one of the most prominent global environmental pollution problems^1,2^. They may originate directly from industrial and personal products, or from the degradation of large-size plastics^3^. For environmental management, we can ban the direct sources of microplastics to a certain extent. However, the wide application of plastic products in daily life makes hundreds of millions of tons of plastic waste, which definitely become the precursors of microplastics, be discharged into the environment each year^4^. As a result, microplastics have been detected in waste water^5,6^, natural water^7,8^, and even in drinking water^9^. At present, the pollution of microplastics has become a persistent environmental problem that needs to be urgently addressed. Therefore, comprehensive and accurate assessment of their environmental risks (e.g., environmental behavior and ecotoxicity) is particularly important for developing effective environmental policies.

Previous studies proved that the large specific surface area makes microplastics show high adsorption capacity to the coexisting organic pollutants, such as polycyclic aromatic hydrocarbons^10^, polychlorinated biphenyls^11^, etc. Some ionizable organic pollutants (e.g., antibiotics) also can be adsorbed on microplastics^12^. The adsorption interaction may further alter the behavior and toxicity of both microplastics and organic pollutants, such as inevitably change the distribution of organic pollutants between the environmental phase and the microplastic phase^13^, or affect the structures and properties of microplastics and organic pollutants and subsequently affect their environmental transformations. More importantly, more organic pollutants can be carried by microplastics into organisms because of the adsorption, which may increase the bioconcentration of chemicals and cause increased toxicity^14,15^. Thus, quantitative measurement of the adsorption for organic pollutions on microplastics is necessary for assessing the environmental risk of both microplastics and organic pollutants in a more comprehensive and accurate way.

Generally, equilibrium partitioning coefficient of organic pollutants between microplastics and water (*K*_d_) is used to represent the adsorption capacity. It can be determined through adsorption equilibrium experiment^11^. Previous studies^12,16^ indicate that the composition and property of both microplastics and water environment media can affect the determined *K*_d_ value. Thus, the specific environmental condition should be considered for measuring the *K*_d_ values, which will greatly increase the amount of experimental work. However, the present research on microplastics is still in its infancy, and the adsorption data is scarce, which will certainly limit their further research on microplastics and their risk assessment. Therefore, there is an urgent need for a fast and accurate method to obtain the *K*_d_ values at different adsorption conditions.

Quantitative structure–property relationship (QSPR) has been proved to be reliable for quickly predicting the properties of chemicals^17,18^. Especially, the polyparameter linear free energy relationship (pp-LFER) models based on Abraham descriptors were widely employed to predict the partitioning of chemicals between two phases and explore the partition mechanisms^19,20^. For example, many researchers predicted the adsorption capacity of polymers with large size (e.g., used for equilibrium passive samplers) based on pp-LFER^21^. However, the large difference in polymer size may limit the application of these already developed models to the prediction of the adsorption capacity for microplastics^22–24^. A few studies established pp-LFER models of log *K*_d_ under corresponding experimental conditions based on their measured experimental values^25–27^. While, the lack of experimental values of Abraham descriptors for many nonpolar chemicals will affect the construction and application of pp-LFER model^20,28^. In order to expand the application range, different descriptors that can be theoretically calculated (e.g., quantum chemical descriptors^29^) may be selected to build the *K*_d_ prediction models. In addition, some ionizable organics such as antibiotics can also be adsorbed by microplastics. The distribution of dissociation species varies under different pH conditions, which will lead to different apparent *K*_d_ values. Thus, the molecular dissociation under certain pH values should be involved in the development of QSPR predictive models.

In this study, we thus collected *K*_d_ values for the three most frequently detected microplastics, including polyethylene (PE), polypropylene (PP) and polystyrene (PS) in different waters, and employed the n-octanol/water distribution coefficient at special pH condition (log *D*), and six quantum chemical descriptors to establish new QSPR models. The main purpose is to develop a more practical computational method that can quickly predict the adsorption capacity of microplastics towards organic pollutants in water environments with different pH values.

## Results and discussion

### QSPR models for the adsorption of PE

Three QSPR models of log *K*_d_ were developed for the adsorption of PE in seawater, freshwater and pure water, respectively:

1 $$\begin{aligned} {\text{Seawater:}}\quad \log K_{{\text{d}}} & = \, \left( {0.725 \, \pm \, 0. \, 058} \right) \, \times \, \log D + \, \left( { - 36.236 \, \pm \, 9.034} \right) \, \times \varepsilon_{\alpha } \\ & \quad + \, \left( { - {23}.{169 } \pm { 4}.{5}0{1}} \right) \, \times \varepsilon_{\beta } + \, \left( {{17}.{856 } \pm { 2}.{572}} \right) \\ \end{aligned}$$

2 $${\text{Freshwater:}}\quad \log K_{{\text{d}}} = \left( {0.667 \, \pm \, 0.047} \right) \times \log D + \, \left( {1.714 \, \pm \, 0.302} \right)$$

3 $${\text{Pure}}\;{\text{water:}}\quad \log K_{{\text{d}}} = \left( {0.449 \, \pm \, 0.041} \right) \times \log D + \, \left( {0.265 \, \pm \, 0.115} \right) \, \times M_{{\text{w}}}^{\prime } \, + \, \left( {1.855 \, \pm \, 0.302} \right)$$

where log *D* is the n-octanol/water distribution coefficient at special pH value, *ε*_α_ is the covalent acidity, *ε*_β_ is the covalent basicity and *M*′_w_ is the relative molecular mass. As shown in Williams plot for model (3) (Fig. S1 of the Supplementary Information, S1), 17α-ethinyl estradiol obtained an absolute *SR* value (− 3.392) larger than 3 and it was diagnosed as an outlier. Structural analysis showed that 17α-ethinyl estradiol is significantly different from other compounds due to its acetylene group and steroidal ring (unsaturated benzene ring connects with saturated six-membered ring). Such discrepancy may be the main cause of predictive inaccuracy. After removing it, the following model was yielded:

4 $${\text{Pure}}\;{\text{water:}}\quad \log K_{{\text{d}}} = \left( {0.486 \, \pm \, 0.035} \right) \times \log D + \, \left( {2.420 \, \pm \, 0.199} \right)$$

**Figure 1:**
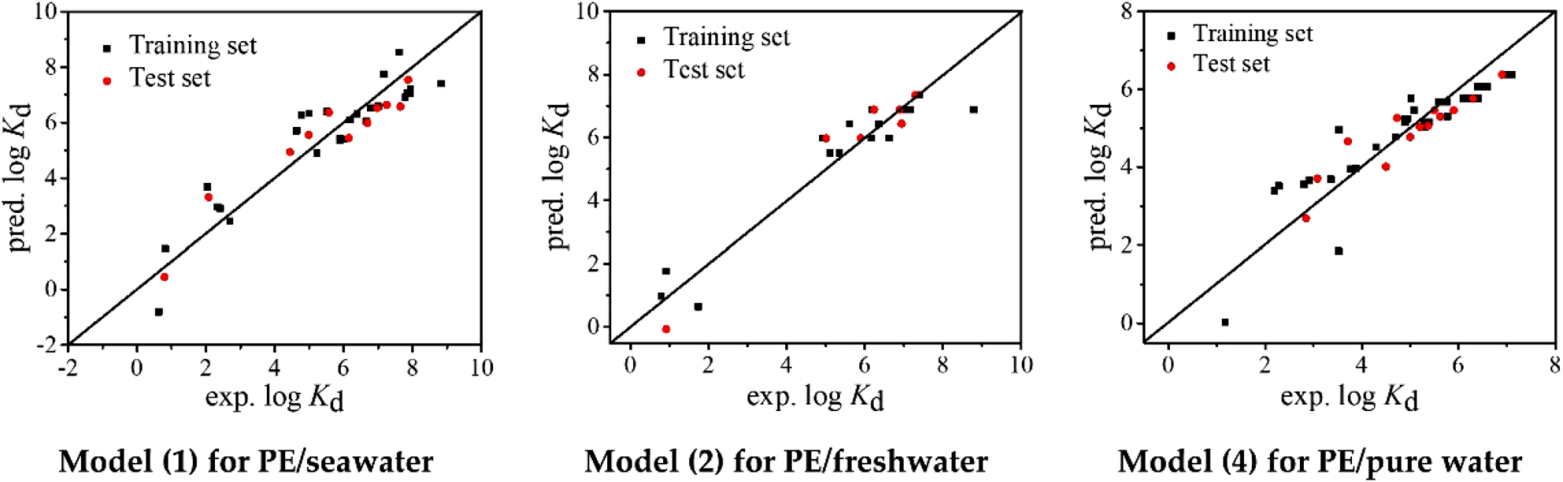
Fitting plots of experimental and predicted log *K*_d_ by models (1), (2) and (4).

**Figure 2:**
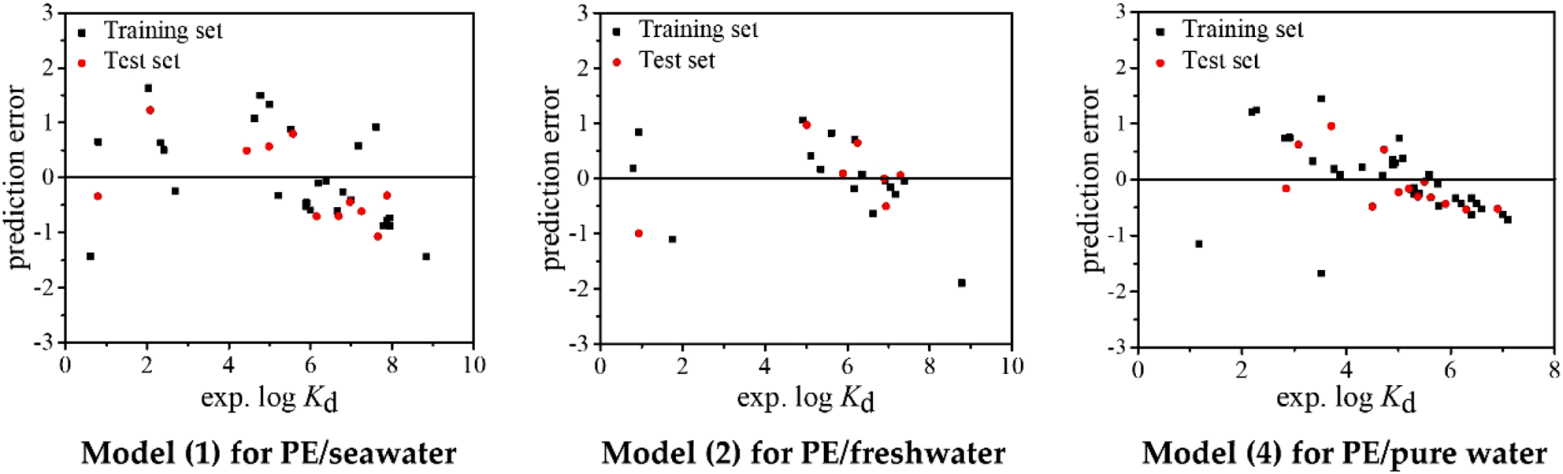
Distributions of prediction errors of log *K*_d_ calculated by models (1), (2) and (4).

The statistical parameters of the developed QSPR models are presented in Table 1. For the models (1), (2) and (4), *R*^2^ = 0.868, 0.903 and 0.811, *Q*^2^ = 0.868, 0.903 and 0.811, and *RMSE* = 0.826, 0.686 and 0.612, respectively. The statistical results indicate that the models have high goodness-of-fit. As shown in Table S1, all the *VIF* values (1.000–1.204) are less than 10, indicating there is no multicollinearity for the three models. The fitting plots (Fig. 1) state a good consistence between the experimental and predicted log *K*_d_ values. As shown in Fig. 2, the distributions of predictive errors show no dependence on experimental log *K*_d_ values. Thus, the developed models have no systematic error, which is also proved by *BIAS* = 0.000–0.001 (Table 1).

**Table 1:** Statistical parameters of the regression models and simulated external validation.

	*N*	*R*^2^	*Q*^2^	*RMSE*	*BIAS*	*MAE*	*MPE*	*MNE*
Model (1)	37	0.868	0.868	0.826	0.000	0.695	1.643	− 1.678
Training set	26	0.857	0.857	0.880	0.000	0.748	1.634	− 1.437
Test set	11	0.902	0.892	0.752	− 0.102	0.664	1.230	− 1.074
Model (2)	24	0.903	0.903	0.686	0.000	0.502	1.044	− 1.983
Training set	17	0.896	0.896	0.732	0.000	0.511	1.059	− 1.895
Test set	7	0.947	0.910	0.661	0.036	0.467	0.970	− 0.998
Model (3)	48	0.800	0.800	0.641	0.000	0.463	2.175	− 1.801
Model (4)	47	0.811	0.811	0.612	0.001	0.470	1.469	− 1.721
Training set	33	0.804	0.804	0.671	0.000	0.522	1.442	− 1.671
Test set	14	0.854	0.835	0.471	− 0.081	0.3838	0.953	− 0.536
Model (5)	35	0.939	0.939	0.381	− 0.003	0.282	1.069	− 0.706
Training set	25	0.945	0.945	0.396	0.000	0.307	0.968	− 0.697
Test set	10	0.898	0.874	0.369	0.047	0.228	0.792	− 0.646
Model (6)	28	0.837	0.837	0.791	0.000	0.634	1.703	− 1.610
Training set	20	0.829	0.829	0.853	0.000	0.669	1.585	− 1.593
Test set	8	0.859	0.843	0.714	0.092	0.654	0.903	− 0.697

For the simulated external validation, the redeveloped QSPR models (S1–S3) based on 70% experimental data and the same descriptors in model (1), (2) and (4) show similar fitting performance (including *R*^2^, *Q*^2^, *RMSE* and *MAE*) and regression coefficients with the models developed by the whole dataset (Table 1). Thus, the models are statistically stable. As the training subsets are randomly assigned, there is no casual correlation. The predictive performance of each rebuilt model to the test set (30% subset, shown by the superscript of b in Table 2) are listed in Table 1. The values of *Q*^2^, *RMSE* and *MAE* indicate excellent predictive quality of the developed QSPR models. The results of leave-one-out cross validation (*Q*^2^_CV_ = 0.882–0.940) also show a good robustness and internal predictivity.

**Table 2:** Experimental and predicted log *K*_d_ values of organic compounds and the values of the selected molecular descriptors in models (1), (2), (4), (5) and (6).

No	Organic compounds	Log *K*_d_^a^		Log *D*	*ε*_*α*_	*ε*_*β*_	*π*	Refs
		Exp.	Pred.					
**For the adsorption of PE in seawater**								
1	2,4,4′-trichlorobiphenyl^b^	6.150	5.490	5.690	0.252	0.317		^11^
2	2,4′,5-trichlorobiphenyl	6.000	5.473	5.690	0.251	0.321		^11^
3	2,2′,3,5′-tetrachlorobiphenyl	5.890	5.444	6.340	0.259	0.329		^11^
4	2,2′,5,5′-tetrachlorobiphenyl	5.900	5.497	6.340	0.257	0.329		^11^
5	2,4,4′,5-tetrachlorobiphenyl	6.660	6.112	6.340	0.246	0.321		^11^
6	2,3′,4,4′-tetrachlorobiphenyl^b^	6.690	6.048	6.340	0.247	0.322		^11^
7	2,2′,4,5,6′-pentachlorobiphenyl	6.190	6.160	6.980	0.248	0.336		^11^
8	2,3,3′,4,4′-pentachlorobiphenyl^b^	6.970	6.561	6.980	0.243	0.325		^11^
9	2,3′,4,4′,5-pentachlorobiphenyl	7.000	6.634	6.980	0.242	0.324		^11^
10	3,3′,4,4′,5-pentachlorobiphenyl	7.780	6.920	6.980	0.235	0.322		^11^
11	3,3′,4,4′,5,5′-hexachlorobiphenyl	8.840	7.450	7.620	0.231	0.327		^11^
12	2,2′,3,4′,5,6-hexachlorobiphenyl	6.790	6.624	7.620	0.248	0.336		^11^
13	2,2′,3,4,4′,5′-hexachlorobiphenyl^b^	7.250	6.686	7.620	0.246	0.335		^11^
14	2,2′,4,4′,5,5′-hexachlorobiphenyl^b^	7.650	6.682	7.620	0.248	0.334		^11^
15	2,3,3′,4,4′,5-hexachlorobiphenyl	7.860	7.135	7.620	0.238	0.329		^11^
16	2,2′,3,3′,4,4′,5-heptachlorobiphenyl	7.940	7.137	8.270	0.245	0.338		^11^
17	2,2′,3,4,4′,5,5′-heptachlorobiphenyl	7.940	7.271	8.270	0.243	0.335		^11^
18	Dichlorodiphenyltrichloroethane^b^	4.986	5.534	5.440	0.238	0.330		^32^
19	Pentachlorobenzene	5.220	4.876	5.220	0.246	0.339		^33^
20	Hexachlorobenzene	4.630	5.669	5.860	0.234	0.344		^33^
21	Phenanthrene^b^	4.440	4.999	4.350	0.254	0.294		^33^
22	Fluoranthene	5.520	6.403	4.930	0.226	0.296		^33^
23	Anthracene	4.770	6.275	4.350	0.230	0.276		^33^
24	Pyrene^b^	5.570	6.413	4.930	0.236	0.279		^33^
25	Chrysene	6.390	6.398	5.520	0.243	0.287		^33^
26	Benzoapyrene	7.170	7.800	6.110	0.226	0.271		^33^
27	Dibenzanthracene^b^	7.870	7.645	6.700	0.235	0.283		^33^
28	Benzo[g,h,i]perylene	7.610	8.656	6.700	0.230	0.246		^33^
29	Pentadecafluorooctanoic acid	2.695	2.673	4.000	0.307	0.300		^13^
30	Dioctyl phthalate	4.993	6.636	8.390	0.283	0.305		^13^
31	Trimethoprim	0.811	1.500	0.730	0.280	0.291		^12^
32	Sulfadiazine^b^	0.797	0.424	− 1.510	0.275	0.274		^12^
33	Oxytetracycline	0.623	− 1.055	− 5.590	0.245	0.258		^34^
34	α-Hexachlorocyclohexane	2.410	2.797	4.260	0.254	0.386		^33^
35	β-Hexachlorocyclohexane	2.040	3.512	4.260	0.237	0.382		^33^
36	γ-Hexachlorocyclohexane	2.330	2.879	4.260	0.257	0.378		^33^
37	δ-Hexachlorocyclohexane^b^	2.080	3.175	4.260	0.244	0.386		^33^
**For the adsorption of PE in freshwater**								
38	2,4,4′-trichlorobiphenyl	5.350	5.509	5.690				^11^
39	2,4′,5-trichlorobiphenyl	5.110	5.509	5.690				^11^
40	2,2′,3,5′-tetrachlorobiphenyl	4.920	5.943	6.340				^11^
41	2,2′,5,5′-tetrachlorobiphenyl^b^	5.010	5.943	6.340				^11^
42	2,4,4′,5-tetrachlorobiphenyl^b^	5.890	5.943	6.340				^11^
43	2,3′,4,4′-tetrachlorobiphenyl	6.170	5.943	6.340				^11^
44	3,3′,4,4′-tetrachlorobiphenyl	6.620	5.943	6.340				^35^
45	2,2′,4,5,6′-pentachlorobiphenyl	5.610	6.370	6.980				^11^
46	2,3,3′,4,4′-pentachlorobiphenyl	6.350	6.370	6.980				^11^
47	2,3′,4,4′,5-pentachlorobiphenyl	6.360	6.370	6.980				^11^
48	3,3′,4,4′,5-pentachlorobiphenyl^b^	6.940	6.370	6.980				^11^
49	3,3′,4,4′,5,5′-hexachlorobiphenyl	8.780	6.797	7.620				^11^
50	2,2′,3,4′,5,6-hexachlorobiphenyl	6.180	6.797	7.620				^11^
51	2,2′,3,4,4′,5′-hexachlorobiphenyl^b^	6.890	6.797	7.620				^11^
52	2,2′,4,4′,5,5′-hexachlorobiphenyl	7.040	6.797	7.620				^11^
53	2,3,3′,4,4′,5-hexachlorobiphenyl	7.170	6.797	7.620				^11^
54	2,2′,3,4,4′,5-hexachlorobiphenyl	6.920	6.797	7.620				^35^
55	2,2′,3,4′,5′,6-hexachlorobiphenyl^b^	6.240	6.797	7.620				^35^
56	2,2′,3,3′,4,4′,5-heptachlorobiphenyl^b^	7.290	7.230	8.270				^11^
57	2,2′,3,4,4′,5,5′-heptachlorobiphenyl	7.390	7.230	8.270				^11^
58	Ciprofloxacin	1.741	0.914	− 1.200				^12^
59	Trimethoprim	0.923	1.967	0.380				^12^
60	Sulfadiazine	0.792	1.234	− 0.720				^12^
61	Amoxicillin^b^	0.924	0.240	− 2.210				^12^
**For the adsorption of PE in pure water**								
62	2,2′,5-trichlorobiphenyl^b^	4.900	5.185	5.690				^36^
63	2,4,4′-trichlorobiphenyl	5.400	5.185	5.690				^36^
64	2,4′,5-trichlorobiphenyl	5.301	5.185	5.690				^37^
65	2,2′,4,4′-tetrachlorobiphenyl	5.083	5.501	6.340				^37^
66	2,2′,5,5′-tetrachlorobiphenyl	5.500	5.501	6.340				^36^
67	2,2′,3,5-tetrachlorobiphenyl	5.500	5.501	6.340				^36^
68	2,3′,4,4′-tetrachlorobiphenyl^b^	5.900	5.501	6.340				^36^
69	2,2′,4,5,5′-pentachlorobiphenyl	6.200	5.812	6.980				^36^
70	2,3,3′,4′,6-pentachlorobiphenyl^b^	6.100	5.812	6.980				^36^
71	2,3′,4,4′,5-pentachlorobiphenyl	6.400	5.812	6.980				^36^
72	2,3,3′,4,4′-pentachlorobiphenyl	6.300	5.812	6.980				^36^
73	2,2′,4,5′,6-pentachlorobiphenyl^b^	5.019	5.812	6.980				^37^
74	2,2′,4,4′,5,5′-hexachlorobiphenyl^b^	6.400	6.123	7.620				^36^
75	2,2′,3,4,4′,5′-hexachlorobiphenyl	6.600	6.123	7.620				^36^
76	2,2′,3,3′,4,5-hexachlorobiphenyl^b^	6.600	6.123	7.620				^36^
77	2,2′,3,3′,4,4′-hexachlorobiphenyl	6.500	6.123	7.620				^36^
78	2,2′,3,4′,5,5′,6-heptachlorobiphenyl	7.100	6.439	8.270				^36^
79	2,2′,3,4,4′,5,5′-heptachlorobiphenyl^b^	7.000	6.439	8.270				^36^
80	2,2′,3,3′,4,4′,5-heptachlorobiphenyl	6.900	6.439	8.270				^36^
81	Chlorobenzene^b^	3.080	3.703	2.640				^30^
82	Benzene	2.190	3.387	1.990				^30^
83	Toluene	2.910	3.654	2.540				^30^
84	Ethyl benzoate	2.810	3.548	2.320				^30^
85	Naphthalene^b^	3.770	3.961	3.170				^30^
86	2-Methylanthracene	5.000	4.797	4.890				^36^
87	1-methylphenanthrene	4.700	4.797	4.890				^36^
88	9,10-Dimethylanthracene^b^	5.300	5.064	5.440				^36^
89	3,6-dimethylphenanthrene	5.200	5.064	5.440				^36^
90	Phenanthrene	4.300	4.534	4.350				^36^
91	Anthracene	4.300	4.534	4.350				^36^
92	Oxytetracycline	1.176	− 0.068	− 5.120				^34^
93	Phenylalanine	3.519	1.798	− 1.280				^38^
94	Cyclohexane	3.880	3.965	3.180				^30^
95	Hexane	4.500	4.019	3.290				^30^
96	Carbamazepine^b^	2.281	3.514	2.250				^39^
97	3-(4-methylbenzylidene)camphor	4.726	5.297	5.920				^39^
98	Triclosan^b^	3.711	4.685	4.660				^39^
99	Sulfamethoxazole	2.845	2.653	0.480				^40^
100	Propanolol^b^	3.362	3.684	2.600				^40^
101	Sertraline	3.522	4.991	5.290				^40^
102	p,p'-DDT	5.590	5.720	6.790				^41^
103	o,p'-DDT	5.760	5.720	6.790				^41^
104	p,p'-DDD	4.890	5.273	5.870				^41^
105	o,p'-DDD	4.940	5.273	5.870				^41^
106	p,p'-DDE	5.770	5.336	6.000				^41^
107	o,p'-DDE	5.620	5.336	6.000				^41^
108	p,p'-DDMU^b^	5.370	5.093	5.500				^41^
**For the adsorption of PP in seawater**								
109	2,3-dichlorobiphenyl^b^	4.980	4.332	5.050		0.321		^13^
110	2,4′-dichlorobiphenyl	4.980	4.411	5.050		0.317		^13^
111	2,4,4′-trichlorobiohenyl	5.090	4.873	5.690		0.317		^13^
112	2,2′,5,5′-tetrachlorobiphenyl	5.090	5.137	6.340		0.329		^13^
113	2,2′,3,5′-tetrachlorobiphenyl	5.140	5.133	6.340		0.329		^13^
114	3,3′,4,4′-tetrachlorobiphenyl	5.630	5.358	6.340		0.318		^13^
115	2,3′,4,4-tetrachlorobiphenyl	5.260	5.277	6.340		0.322		^13^
116	2,3′,4,4′,5-pentachlorobiphenyl^b^	5.710	5.708	6.980		0.324		^13^
117	2,3,3′,4,4′-pentachlorobiphenyl^b^	5.770	5.690	6.980		0.325		^13^
118	2,2′,3,4′,5-pentachlorobiphenyl	5.510	5.526	6.980		0.334		^13^
119	2,2′,3,5′,6-pentachlorobiphenyl^b^	5.260	5.577	6.980		0.331		^13^
120	2,3,3′,4′,6-pentachlorobiphenyl	5.630	5.538	6.980		0.333		^13^
121	2,2′,4,5,5′-pentachlorobiphenyl^b^	5.510	5.594	6.980		0.330		^13^
122	2,2′,3,3′,4,6′-hexachlorobiphenyl^b^	6.190	5.994	7.620		0.335		^13^
123	2,3,3′,4,5,6-hexachlorobiphenyl^b^	6.060	5.993	7.620		0.335		^13^
124	2,2′,4,4′,5,5′-hexachlorobiphenyl	6.190	6.013	7.620		0.334		^13^
125	2,2′,3,4,4′,5-hexachlorobiphenyl	5.770	5.977	7.620		0.335		^13^
126	2,2′,3,3′,4,4′-hexachlorobiphenyl	5.450	5.930	7.620		0.338		^13^
127	2,2′,3,4′,5,5′,6-heptachlorobiphenyl^b^	5.730	6.448	8.270		0.336		^13^
128	Pentachlorobenzene	4.500	4.098	5.220		0.339		^33^
129	Hexachlorobenzene	5.010	4.489	5.860		0.344		^33^
130	Phenanthrene	4.000	4.314	4.350		0.294		^33^
131	Fluoranthene^b^	4.790	4.720	4.930		0.296		^33^
132	Anthracene	4.290	4.678	4.350		0.276		^33^
133	Pyrene	4.800	5.038	4.930		0.279		^33^
134	Chrysene	5.510	5.344	5.520		0.287		^33^
135	Benzoapyrene	6.100	6.079	6.110		0.271		^33^
136	Dibenz[a,h]anthracene	7.000	6.294	6.700		0.283		^33^
137	Benzo[g,h,i]perylene	6.690	7.006	6.700		0.246		^33^
138	Trimethoprim	0.594	1.663	0.730		0.291		^12^
139	Sulfadiazine	0.853	0.299	− 1.510		0.274		^12^
140	α-Hexachlorocyclohexane	2.690	2.474	4.260		0.386		^33^
141	β-Hexachlorocyclohexane	2.180	2.554	4.260		0.382		^33^
142	γ-Hexachlorocyclohexane^b^	2.580	2.633	4.260		0.378		^33^
143	δ-Hexachlorocyclohexane	2.230	2.483	4.260		0.386		^33^
**For the adsorption of PS in seawater**								
144	Pentachlorobenzene	5.100	4.070	5.220			1.138	^33^
145	Hexachlorobenzene^b^	5.280	4.545	5.860			1.204	^33^
146	Phenanthrene	5.390	5.190	4.350			1.518	^33^
147	Fluoranthene^b^	5.910	5.528	4.930			1.553	^33^
148	Anthracene	5.610	5.560	4.350			1.616	^33^
149	Pyrene	5.840	6.437	4.930			1.794	^33^
150	Chrysene^b^	6.630	6.146	5.520			1.661	^33^
151	Benzo[a]pyrene	6.920	7.347	6.110			1.924	^33^
152	Dibenz[a,h]anthracene	7.520	7.267	6.700			1.847	^33^
153	Benzo[g,h,i]perylene	7.150	5.540	6.700			1.388	^33^
154	4-Fluorobenzoic acid	2.134	1.771	− 0.940			1.112	^16^
155	Trimethoprim	0.863	2.566	0.730			1.164	^12^
156	Sulfadiazine	0.833	1.874	− 1.510			1.193	^12^
157	α-Hexachlorocyclohexane	3.190	3.297	4.260			1.024	^33^
158	β-Hexachlorocyclohexane^b^	2.630	3.515	4.260			1.082	^33^
159	γ-Hexachlorocyclohexane	3.010	3.416	4.260			1.056	^33^
160	δ-Hexachlorocyclohexane	2.800	3.221	4.260			1.004	^33^
161	Perfluoropentanoic acid	2.432	0.835	1.540			0.628	^16^
162	Perfluorohexanoic acid^b^	1.760	1.181	2.220			0.655	^16^
163	Perfluoroheptanoic acid	1.731	1.492	3.110			0.654	^16^
164	Perfluorodecanoic acid	2.669	2.480	5.780			0.663	^16^
165	Pentadecafluorooctanoic acid	3.220	2.055	4.000			0.719	^16^
166	Heptadecafluorooctanesulfonamide	2.147	2.963	5.800			0.789	^16^
167	Perfluoro-1-octanesulfonyl fluoride^b^	2.792	3.233	6.890			0.758	^16^
168	Perfluoroundecanoic acid	2.752	2.992	6.670			0.715	^16^
169	Perfluorododecanoic acid^b^	2.720	3.308	7.550			0.715	^16^
170	Pentacosafluorotridecanoic acid^b^	3.162	3.558	8.440			0.697	^16^
171	Perfluorotetradecanoic acid	3.088	3.904	9.330			0.704	^16^

^a^The unit of *K*_d_ is kg/L; ^b^ The compounds used for test subset in simulated external validation.

**Figure 3:**
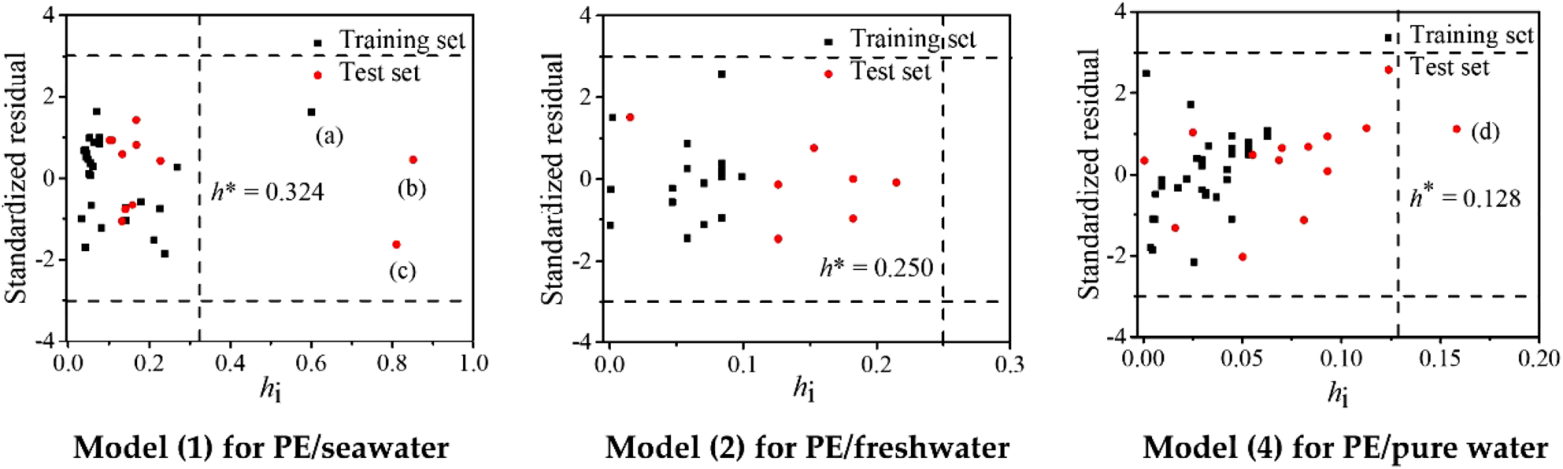
Williams plots for the applicability domain of models (1), (2) and (4). The *h*_*i*_ refers to the verse leverage value. (**a**) oxytetracycline; (**b**) sulfadiazine; (**c**) δ-hexachlorocyclohexane; (**d**) 2,2′,3,3′,4,4′,5-heptachlorobiphenyl.

Williams plots were employed to test the application domain of the QSPR models (1), (2) and (4). The calculated alert value h^*^ are 0.324, 0.250 and 0. 128, respectively. As shown in Fig. 3, there are three (oxytetracycline, sulfadiazine and δ-hexachlorocyclohexane), and one (2,2′,3,3′,4,4′,5-heptachlorobiphenyl) compounds located at the right side of h^*^ for models (1) and (4), respectively. As their absolute *SR* values are < 3, these chemicals are not diagnosed to be outliers. In summary, these results indicate the developed QSPR models have excellent generalization capabilities in their descriptor matrix. Given the molecular structures for developing models, QSPR model (1) can be used to predict the log *K*_d_ values of organics including polychlorinated biphenyls, antibiotics, polycyclic aromatic hydrocarbons, chlorobenzenes, perfluorinated compounds and hexachlorocyclohexanes between PE and sea water; model (2) can be employed for predicting the log *K*_d_ values of polychlorinated biphenyls and antibiotics between PE and fresh water; model (4) can be performed to predict the adsorption of PE in pure water towards organic pollutants such as polychlorinated biphenyls, antibiotics, polycyclic aromatic hydrocarbons, chlorobenzenes, aromatic hydrocarbons and aliphatic hydrocarbons.

The n-octanol/water distribution coefficient at special pH value (log *D*) was selected for all the three log *K*_d_ predictive models for PE in seawater, freshwater and pure water. The experimental log *K*_d_ values significantly correlate with log *D*, which yields positive correlation coefficients (0.725, 0.667 and 0.486) in models (1), (2) and (4). Thus, the organic pollutants with high hydrophobicity will prefer to be adsorbed onto the PE. For example, hydrophobic polychlorinated biphenyls (PCBs) with large log *D* values exhibit higher log *K*_d_ values than ionizable organic pollutants (e.g., antibiotics). This is because the hydrophobicity of PE itself makes hydrophobic interaction as the main mechanism in the adsorption of PE towards organic pollutants. The same adsorption mechanism was also confirmed by Hüffer et al. who established prediction model based on the log *K*_ow_ values of seven organic compounds^30^.

For the adsorption of PE in seawater, *ε*_*α*_ and *ε*_*β*_, which respectively represents covalent acidity and covalent basicity, were also selected. The quantum chemical descriptor of *ε*_*α*_ shows a negative contribution to the log *K*_d_ values, suggesting that organic pollutant with large *ε*_*α*_ value prefers to dissolve in water, leading to a decrease in log *K*_d_. That means the surface of PE has a weaker H-accepting ability to organic pollutants than water at the adsorption interface^31^. Similarly, the log *K*_d_ values increase with decreasing *ε*_*β*_, indicating that the H-donating ability of the PE surface is also weaker than water. It follows that hydrogen bond interaction is also an important mechanism for the interactions between PE and organic pollutants in sea water.

Compared with fresh water and pure water, the high salinity of seawater can enhance the dipole–dipole and dipole–induced dipole interactions in the system, which can make hydrogen bonds form easily. As a result, *ε*_*α*_ and *ε*_*β*_ play more important role in the log *K*_d_ value of PE for seawater. In brief, the distribution behavior of the studied organics between PE and water is mainly affected by the hydrophobic interaction. For the adsorption in seawater, hydrogen bond interaction is another important driving force.

### QSPR model for the adsorption of PP

A QSPR model of log *K*_d_ was yielded for the adsorption of PP in seawater:

5 $${\text{Seawater:}}\quad \log K_{{\text{d}}} = \left( {0.751 \pm 0. \, 035} \right) \times \log D + \left( { - 19.323 \pm 2.072} \right) \times \varepsilon_{\beta } + \left( {6.735 \pm 0.663} \right)$$

Values of *R*^2^, *Q*^2^, and *RMSE* are 0.939, 0.939 and 0.381, respectively. Thus, the model (5) show great goodness of fitting and can explain 94% variability of the whole dataset. The nonlinearity of model (5) has been proved by the *VIF* values (1.034 for both descriptors, Table S1). As shown in Fig. S2, the predicted log *K*_d_ values show good consistence with their experimental values. The Fig. S3 and *BIAS* value (− 0.003) proved that there is no dependence of predictive errors on experimental log *K*_d_ values.

For the simulated external validation, the regression coefficients (*R*^2^ = 0.945, *RMSE* = 0.396 and *MAE* = 0.307) and statistical parameters of the training subset are similar to that of the whole dataset (Table 1 and model S4). Thus, model (5) is statistically stable and there is no casual correlation. As shown in Table 1, the high prediction quality of the developed QSPR model can be proved by the predictive performance of the new model (*Q*^2^ = 0.874, *RMSE* = 0.369 and *MAE* = 0.228) to the test subset. Furthermore, model (5 has good robustness and internal predictive ability (*Q*^2^_CV_ = 0.957). The Williams plot for the applicability domain of model (5) (Fig. S4) shows that there are two compounds (sulfadiazine and *γ*-hexachlorocyclohexane) located at the right side of h^*^ (0.257). While, these two compounds yield absolute *SR* values < 3, indicating they are not outliers. Thus, model (5) can be used to predict the log *K*_d_ values of PE in seawater towards the organics including polychlorinated biphenyls, chlorobenzenes, hexachlorocyclohexanes, polycyclic aromatic hydrocarbons and antibiotics.

For the adsorption of PP in sea water, log *D* and *ε*_*β*_ were also selected in model (5). Thus, hydrophobic interaction and hydrogen bond interaction also play determining roles in the adsorption. However, unlike the log *K*_d_ predictive model of PE in seawater, the *ε*_*α*_ representing the covalent acidity is not selected in model (5). Such dissimilarity may come from the addition of methyl groups in the PP structure that reduces the difference of H-accepting ability between the microplastics and water, consequently resulting in a negligible contribution of *ε*_*α*_ in the adsorption of PP.

### QSPR model for the adsorption of PS

For the adsorption of PS in seawater, the experimental log *K*_d_ values of 28 organic pollutants (of which 14 are ionizable compounds) were used to established predictive model:

6 $${\text{Seawater:}}\quad \log K_{{\text{d}}} = \left( {0.357 \pm 0. \, 062} \right) \times \log D + \left( {3.766 \pm 0.384} \right) \times \pi + \left( { - 2.080 \pm 0.540} \right)$$

As shown in Tables 1 and S1, the obtained statistical parameters (*R*^2^ = *Q*^2^ = 0.837) prove a good regression performance and the calculated *VIF* values (1.000 for both descriptors) prove no multicollinearity of model (6). Meanwhile, the favorable consistence between the experimental and predicted log *K*_d_ values was observed in Fig. S5. The pattern of predictive errors shown in Fig. S6 reveals no systematic error for model (6), which is also verified by *BIAS* = 0.000 (Table 1).

Based on the training subset (70%), similar regression coefficients and statistical parameters of the new model (S5) were obtained (Table 1). The comparable statistics were also received for the test set. Moreover, *Q*^2^_CV_ value (0.898) of the leave-one-out cross validation was obtained, higher than the acceptable criteria. Thus, model (6) has satisfactory robustness and internal predictive ability. As shown in the Fig. S7 of Williams plot, three compounds (fluoranthene, chrysene and pentacosafluorotridecanoic acid) with ׀SR׀ < 3 locate at the right side of h^*^ (0.321), indicating that they are not outliers. In conclusion, model (6) can be employed for predicting the adsorption carrying capacity (log *K*_d_) of PS for organic pollutants (especially for ionizable organic pollutants) within the application domain in seawater. In previous study^20^, the influence of dissociation on log *K*_d_ for ionizable organic pollutants was not considered in the construction of predictive models. In fact, the physicochemical properties (e.g., hydrophobicity) of various dissociation species are quite different, which may significantly affect the partition of ionizable organic pollutants between PS and seawater. Therefore, the predictive models established without considering the effect of pH on the distribution of dissociation species is only applicable to predict log *K*_d_ values under the experimental water pH. However, the QSPR model (6) constructed in this study can expand the predictive application to various pH values. Limited by the number of ionizable compounds and pH range used for model construction, the developed models are more suitable for the pH range of natural waters (6–9).

The presence of log *D* in model (6) proves that hydrophobic interaction also can enhance the adsorption of organics on PS in seawater. In addition to log *D*, *π* was also selected. The experimental log *K*_d_ values positively correlate with *π* (3.766) in the QSPR model, indicating that chemicals with larger *π* value preferred to be adsorbed onto PS in seawater. As shown in Tables 2 and S2, the organic compound, which contains strong π–electron conjugation in the structure, generally has a large *π* value. Thus, it can be inferred that the π − π interaction also contributes to the adsorption for PS. The phenyl groups in the PS structure produce higher π–π interactions with organic chemicals than PE and PP, thus yielding higher log *K*_d_ values (Table 2). For example, the log *K*_d_ value of phenanthrene onto PS (5.50) is much higher than that on PE (4.440) and PP (4.000) in sea water. In brief, hydrophobic interaction and π–π interaction play important roles in the adsorption of PS in sea water.

## Materials and methods

### Collection of experimental ***K***_d_ values

In order to improve the predictive accuracy, the properties of microplastics and water environment media were considered by screening and classifying the experimental data used for modeling. For the adsorption of organic pollutants on PE, 37, 24 and 48 experimental *K*_d_ values were collected for seawater, freshwater, and pure water, respectively. For the adsorption of PP and PS in seawater, 35 and 28 experimental *K*_d_ values were selected, respectively. All these collected data are listed in Table 2. The unit of all *K*_d_ values was unified to kg/L. As the value of *K*_d_ is quite large, its logarithmic form (log *K*_d_) was used for developing QSPR models. Experimental conditions for determining *K*_d_ values are shown in Table S3. Molecular structures for all organic pollutants, including polychlorinated biphenyls, polycyclic aromatic hydrocarbons, aromatic hydrocarbons, chlorobenzenes, hexachlorocyclohexanes, aliphatic hydrocarbons, antibiotics and perfluorinated compounds, are shown in Table S2.

### Molecular structural parameters

Based on the previous studies^20,30^, hydrophobic interaction, hydrogen bond and π-π interaction may play important roles in the adsorption of microplastics towards organic pollutants. Thus, the n-octanol/water distribution coefficient at special pH value (log *D*), molecular mass (*M*′_w_ = *M*_w_/100) and six quantum chemical descriptors were calculated for developing QSPR models (Table S4). Six selected quantum chemical descriptors include molecular volume (*V*′ = *V*/100), the ratio of average molecular polarizability and molecular volume (*π* = *α*/*V*), the most positive atomic charge on H atom (*q*H^+^), the most negative atomic charge (*q*^−^), covalent acidity (*ε*_α_ = *E*_LUMO_ − *E*_HOMO-water_), and covalent basicity (*ε*_β_ = *E*_LUMO-water_ − *E*_HOMO_) where *E*_HOMO_ refers to the highest occupied molecular orbital energy and *E*_LUMO_ stands for the lowest unoccupied molecular orbital energy. For non-dissociable compounds, the n-octanol/water distribution coefficients are the same for the different pH values. While for the ionizable organics, different log *D* values for the relevant experimental conditions were obtained from SciFinder^42^. The values of *M*_w_, *V*, *π*, *q*^+^, *q*^–^, *E*_HOMO_ and *E*_LUMO_ were extracted from the Gaussian output files.

The structures of all the molecules were optimized at B3LYP/6-31G(d,p) level using Gaussian 09 program package^43^, and confirmed to be local minima by vibrational frequency analyses with the same method. For the ionizable compounds, all dissociation species may exist under the experimental pH conditions were optimized. The apparent value of each quantum chemical descriptor at special pH value can be calculated as:

7 $$X_{{{\text{pH}}}} = \, \sum \alpha_{i} X_{i}$$

where *X* stands for the quantum chemical descriptor, *α*_*i*_ is the fraction of each dissociation species under the experimental pH conditions (Table S3), which can be calculated through the p*K*_a_ values of the ionizable compounds (Table S5).

### Model development and validation

The initial prediction model can be expressed as follows:

8 $${\log}K_{{\text{d}}} = d\log D + vV^{\prime } \, + mM_{{\text{w}}}^{\prime } \, + a\varepsilon_{\alpha } + b\varepsilon_{\beta } + p\pi + fq^{ + } + eq^{-} + g$$

where *d*, *v*, *m*, *a*, *b*, *p*, *f* and *e* are fitting coefficients, and *g* is a regression constant. The model development and variable filtration were performed by multiple linear regression (MLR)^44^ with a step-wise algorithm embedded in soft package SPSS 21.0. The statistical parameters squared correlation coefficient (*R*^2^) and root-mean-square error (*RMSE*) were calculated to characterize the fitting performance and predictive squared correlation coefficient (*Q*^2^) was used to represent the predictive ability of the developed QSPR models^45^. Statistically, the values of *R*^2^ and *Q*^2^ should be > 0.5. The larger value of *Q*^2^ indicates the predictive ability of model is stronger. The collinearity of the employed parameters was assessed by the variance inflating factor (*VIF*) values. The calculation details for all statistical parameters were listed in the Text S1.

The statistical robustness and predictive ability of the developed models were verified by the simulated external validation and leave-one-out cross validation^46^. The data set was randomly divided into a 70% training set and a 30% test subset^25,29^ (shown in Table 2). Based on the training set, a new model was rebuilt with the same descriptors selected by the whole dataset. Subsequently, log *K*_d_ values in the test subset were predicted and evaluated by the new models. The values of *R*^2^, *Q*^2^ and *RMSE* of the simulated external validation were calculated to estimate the predictive performance^47^. To assess the model robustness, cross-validated correlation coefficients (*Q*^2^_CV_) were calculated with Weka 3.8.0^48^.

### Outliers and application domain

The Williams plot was performed to visualize the application domain and determine the outliers^49,50^, where the leverage value (*h*_i_) was set as horizontal coordinate and standardized predictive residuals (*SR*) was set as vertical coordinate. Hat-matrix was used to calculate the *h*_i_ values^51^. When the absolute value of *SR* is larger than 3, the relevant compound was designated as outlier and should be removed. Warning value (h^*^) is defined as h* = 3*p*/*n*^51^, where *p* and *n* are the number of descriptors and compounds in the developed model, respectively. If *h*_i_ > h^*^, the compound is far away from the descriptor-matrix center. Thus, the Williams plot also can be used to describe the distribution of chemicals in the whole descriptor matrix.

## Conclusions

QSPR models were established for predicting the adsorption capacity of organic pollutants on PE in seawater, freshwater and pure water, on PP in seawater and on PS in seawater. The statistical results and application domain validations indicate the satisfactory goodness-of-fit, robustness and predictive ability of the predictive models. The constructed models have two significant advantages: (1) the descriptors used in the models are not dependent on experimental values and can be simply obtained based on the structure of organic pollutants; (2) the models can be used to predict the log *K*_d_ values of ionizable compounds at various pH values.

Based on the descriptors selected in the predictive models, main adsorption mechanisms between microplastics and organic pollutants were explored. For all the systems studied here, hydrophobic interaction has been proved to be an indispensable factor for the adsorption. Hydrogen bond interaction and π–π interaction are also considerable mechanisms for the adsorption onto PE and PP in sea water and the adsorption onto PS in sea water, respectively. Thus, this study provides us feasible tools to rapidly and easily predict the adsorption capacity of organic pollutants onto different microplastics in various waters, and also reveals the possible adsorption mechanisms. It will be helpful for further investigation of the environmental risks of both microplastics and their coexisting organic pollutants. Of course, the application scope of the predictive models constructed in this study is still limited as the limitation of experimental data. Therefore, it is still necessary to develop QSPR models for other types of microplastics in the further, or develop predictive method that does not depend on experimental data.

## Supplementary information

Supplementary information
