# Supplementary material for: QSPR models for predicting the adsorption capacity for microplastics of polyethylene, polypropylene and polystyrene
Source: Sci Rep. 2020 Sep 3;10:14597. doi: 10.1038/s41598-020-71390-3 (PMC7473759; doi:10.1038/s41598-020-71390-3)
Supplement: Supplementary file 1 — Supplementary information [file 41598_2020_71390_MOESM1_ESM.pdf]

Supplementary Information

QSPR models for predicting the adsorption capacity for microplastics of polyethylene, polypropylene and polystyrene

Miao Li, Haiying Yu, Yifei Wang, Jiagen Li, Guangcai Ma and Xiaoxuan Wei*

College of Geography and Environmental Sciences, Zhejiang Normal University, Yingbin Avenue 688, 321004, Jinhua, P.R. China

***** Correspondence: xxwei@zjnu.edu.cn; Tel.: 0086 579 8228 2273

Contents:

Models (S1)~(S10): Redeveloped models of the training and test subsets.
Text S1: Computational details of the statistical parameters.
Table S1: Coefficients, *VIF*, *t* and *p* values of the descriptors involved in log K_d models.
Table S2: Molecular structures of all the studied organic compounds.
Table S3: Experimental conditions of log K_d values and size of microplastics.
Table S4: Values of log *D*, molecular mass and six quantum chemical descriptors.
Table S5: Dissociation information of all dissociable compounds.
Fig. S1: Williams plots for the applicability domain of model (3).
Fig. S2: Fitting plots of experimental and predicted log K_d values by model (5).
Fig. S3: Distributions of prediction errors of log K_d calculated by model (5).
Fig. S4: Williams plot for the applicability domain of model (5).
Fig. S5: Fitting plots of experimental and predicted log K_d values by model (6).
Fig. S6: Distributions of prediction errors of log K_d calculated by model (6).
Fig. S7: Williams plot for the applicability domain by model (6).

The redeveloped models of the training sets are listed as following:

$$\log K_d = (0.690 \pm 0.070) \times \log D + (-39.852 \pm 10.281) \times \varepsilon_\alpha + (-21.286 \pm 5.650) \times \varepsilon_\beta + (18.311 \pm 2.958) \quad \text{(S1)}$$

$$\log K_d = (0.708 \pm 0.062) \times \log D + (1.491 \pm 0.397) \quad \text{(S2)}$$

$$\log K_d = (0.474 \pm 0.042) \times \log D + (2.455 \pm 0.242) \quad \text{(S3)}$$

$$\log K_d = (0.772 \pm 0.041) \times \log D + (-19.028 \pm 2.337) \times \varepsilon_\beta + (6.536 \pm 0.742) \quad \text{(S4)}$$

$$\log K_d = (0.389 \pm 0.078) \times \log D + (3.511 \pm 0.489) \times \pi + (-1.922 \pm 0.657) \quad \text{(S5)}$$

The regression models of the test sets are listed as following:

$$\log K_d = (1.073 \pm 0.085) \times \log D + (-29.572 \pm 4.537) \times \varepsilon_\beta + (37.615 \pm 18.712) \times \varepsilon_\alpha + (-0.142 \pm 5.061) \quad \text{(S6)}$$

$$\log K_d = (0.591 \pm 0.062) \times \log D + (2.142 \pm 0.420) \quad \text{(S7)}$$

$$\log K_d = (0.543 \pm 0.065) \times \log D + (2.176 \pm 0.360) \quad \text{(S8)}$$

$$\log K_d = (0.615 \pm 0.083) \times \log D + (-24.109 \pm 5.508) \times \varepsilon_\beta + (9.203 \pm 1.959) \quad \text{(S9)}$$

$$\log K_d = (0.245 \pm 0.118) \times \log D + (4.374 \pm 0.581) \times \pi + (-2.089 \pm 1.013) \quad \text{(S10)}$$

Text S1 The computational formulations of the squared correlation coefficient R^2, predictive squared correlation coefficient Q^2, root-mean-square error (*RMSE*) and variance inflating factor (*VIF*) are shown here. The two statistics are used to quantify the validation and prediction performance of the developed QSPR models.

$$R^2 = 1 - \frac{\sum_{i=1}^{n}(y_i^{fit} - y_i^{exp})^2}{\sum_{i=1}^{n}(y_i^{exp} - \bar{y}^{exp})^2} \quad \text{(S11)}$$

$$Q^2 = 1 - \frac{\sum_{i=1}^{n}(y_i^{pred} - y_i^{exp})^2}{\sum_{i=1}^{n}(y_i^{exp} - \bar{y}^{exp})^2} \quad \text{(S12)}$$

$$RMSE = \sqrt{\frac{\sum_{i=1}^{n}(y_i^{pred} - y_i^{exp})^2}{n}} \quad \text{(S13)}$$

$$VIF = \frac{1}{1 - R_i^2} \quad \text{(S14)}$$

where, y_i^{fit}, y_i^{exp}, $\bar{y}^{exp}$, and y_i^{pred} is the regression-fitted, experimental, average experimental, and predictive value of logK_d, respectively. R_i^2 is the determination coefficient for the regression of one parameter on all other independent variables in the dataset.

In statistics, the mean absolute error (*MAE*) is a quantity used to measure how close the predictive values are to the experimental values. The mean absolute error can be calculated by:

$$MAE = \frac{1}{n}\sum_{i=1}^{n}|E_i|, \quad |E_i| = |y_i^a - y_i^b| \quad \text{(S15)}$$

where, the y_i^a is the predictive value and the y_i^b is the experimental values.

A systematic error is an error that will occur consistently in only direction each time the experiment is performed and the values of the measurement will always be greater or lesser than the real values. Systematic errors most commonly arise from defects in the instrumentation or from using improper measuring techniques. The systematic error can be calculated by:

$$BIAS = \frac{1}{n}\sum_{i=1}^{n}E_i, \quad E_i = y_i^a - y_i^b \quad \text{(S16)}$$

where, the y_i^a is the predictive value and the y_i^b is the experimental values.

Table S1 Coefficients, *t* value of the *t* test, significance level (*p* value) and variance inflation factor (*VIF*) of the molecular structural descriptors involved in log K_d models.

Models	Parameters	Coefficients	*t* value	*p* value	*VIF*
Model (1)	log *D*	0.725	12.517	<0.001	1.204
	ε_β	−23.169	−5.147	<0.001	1.123
	ε_α	−36.236	−4.011	<0.001	1.083
Model (2)	log *D*	0.667	14.333	<0.001	1.000
Model (3)	log *D*	0.449	10.936	<0.001	1.229
	M_w	0.265	2.306	<0.05	1.229
Model (4)	log *D*	0.486	13.901	<0.001	1.000
Model (5)	log *D*	0.751	21.401	<0.001	1.034
	ε_β	−19.323	−9.326	<0.001	1.034
Model (6)	π	3.766	9.815	<0.001	1.000
	log *D*	0.357	5.718	<0.001	1.000

Table S2 Molecular structures and CAS number of all the studied organic compounds

No.	Organic pollutants	CAS	Structures
1	2,3-dichlorobiphenyl	16605-91-7	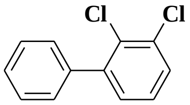
2	2,4'-dichlorobiphenyl	34883-43-7	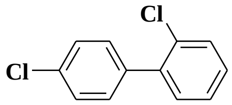
3	2,4,4'-trichlorobiphenyl	7012-37-5	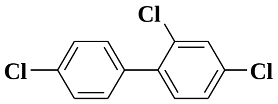
4	2,4',5-trichlorobiphenyl	16606-02-3	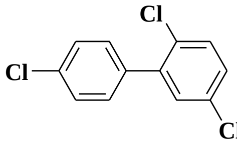

No.	Organic pollutants	CAS	Structures
5	2,2',5-trichlorobiphenyl	37680-65-2	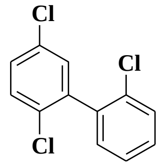
6	2,2',3,5'-tetrachlorobiphenyl	41464-39-5	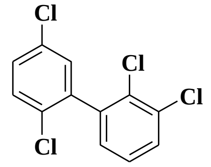
7	2,2',5,5'-tetrachlorobiphenyl	35693-99-3	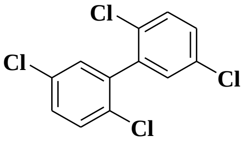
8	2,4,4',5-tetrachlorobiphenyl	32690-93-0	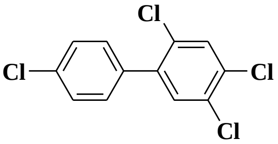
9	2,3',4,4'-tetrachlorobiphenyl	32598-10-0	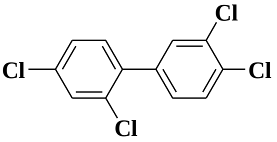
10	3,3',4,4'-tetrachlorobiphenyl	32598-13-3	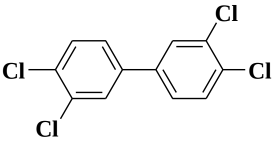
11	2,2',3,5-tetrachlorobiphenyl	70362-46-8	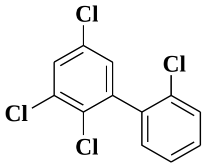
12	2,2',4,4'-tetrachlorobiphenyl	2437-79-8	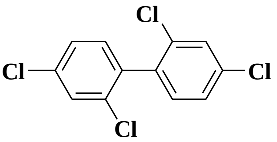
13	2,2',4,5,6'-pentachlorobiphenyl	68194-06-9	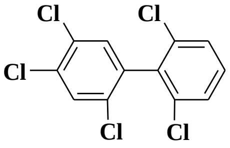
14	2,3,3',4,4'-pentachlorobiphenyl	32598-14-4	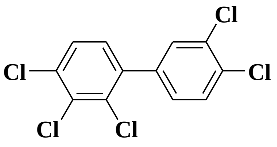

No.	Organic pollutants	CAS	Structures
15	2,3',4,4',5-pentachlorobiphenyl	31508-00-6	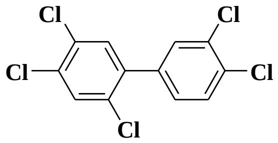
16	3,3',4,4',5-pentachlorobiphenyl	57465-28-8	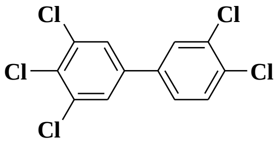
17	2,2',4,5,5'-pentachlorobiphenyl	37680-73-2	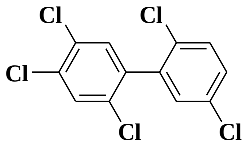
18	2,3,3',4',6-pentachlorobiphenyl	38380-03-9	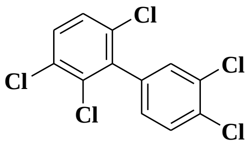
19	2,2',3,4',5-pentachlorobiphenyl	68194-07-0	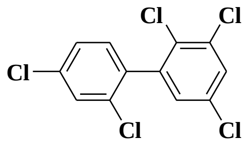
20	2,2',3,5',6-pentachlorobiphenyl	38379-99-6	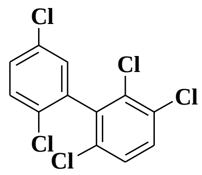
21	2,2',4,5',6-pentachlorobiphenyl	60145-21-3	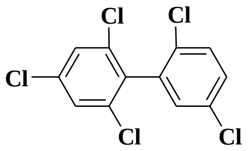
22	2,2',3,4',5,6-hexachlorobiphenyl	68194-13-8	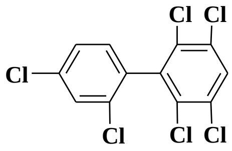
23	2,2',3,4,4',5'-hexachlorobiphenyl	35065-28-2	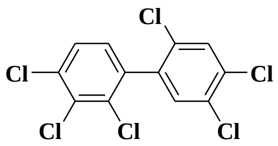
24	2,2',4,4',5,5'-hexachlorobiphenyl	35065-27-1	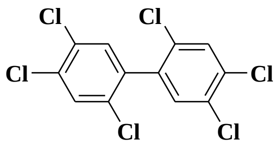

No.	Organic pollutants	CAS	Structures
25	2,3,3',4,4',5-hexachlorobiphenyl	38380-08-4	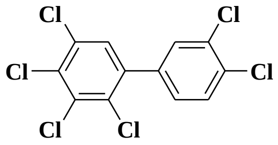
26	3,3',4,4',5,5'-hexachlorobiphenyl	32774-16-6	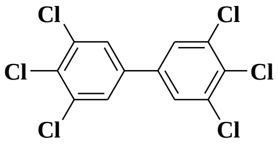
27	2,2',3,4,4',5-hexachlorobiphenyl	35694-06-5	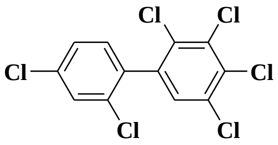
28	2,2',3,4',5',6-hexachlorobiphenyl	38380-04-0	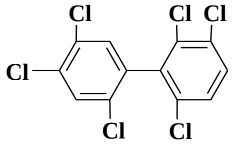
29	2,2',3,3',4,5-hexachlorobiphenyl	55215-18-4	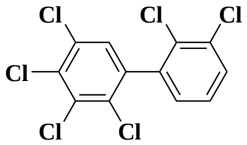
30	2,2',3,3',4,4'-hexachlorobiphenyl	38380-07-3	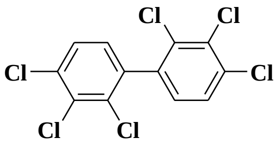
31	2,2',3,3',4,6'-hexachlorobiphenyl	38380-05-1	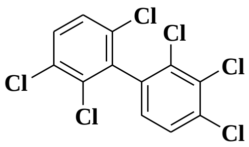
32	2,3,3',4,5,6-hexachlorobiphenyl	41411-62-5	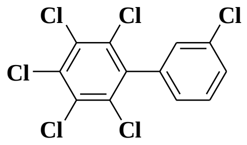
33	2,2',3,3',4,4',5-heptachlorobiphenyl	35065-30-6	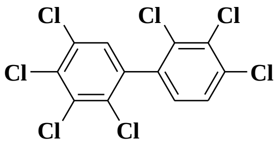
34	2,2',3,4,4',5,5'-heptachlorobiphenyl	35065-29-3	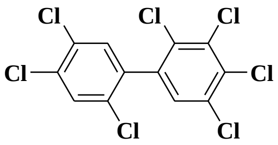

No.	Organic pollutants	CAS	Structures
35	2,2',3,4',5,5',6-heptachlorobiphenyl	52663-68-0	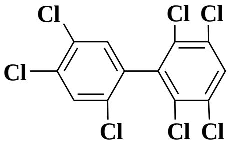
36	Dichlorodiphenyltrichloroethane	3547-04-4	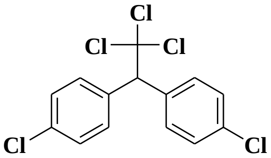
37	Chlorobenzene	108-90-7	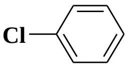
38	Pentachlorobenzene	608-93-5	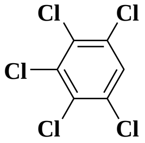
39	Hexachlorobenzene	118-74-1	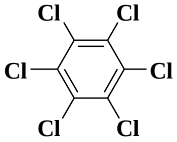
40	Benzene	71-43-2	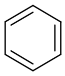
41	Toluene	108-88-3	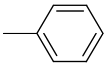
42	Naphthalene	91-20-3	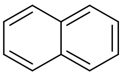
43	2-Methylanthracene	613-12-7	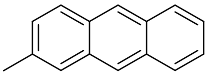
44	1-methylphenanthrene	832-69-9	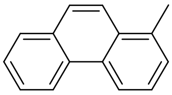
45	9,10-Dimethylanthracene	781-43-1	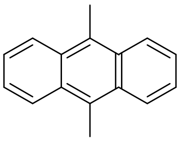
46	3,6-dimethylphenanthrene	1576-67-6	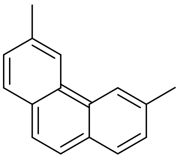
47	Phenanthrene	85-01-8	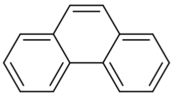
48	Anthracene	120-12-7	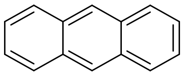

No.	Organic pollutants	CAS	Structures
49	Pyrene	129-00-0	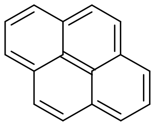
50	Fluoranthene	206-44-0	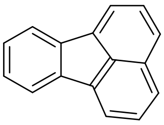
51	Chrysene	218-01-9	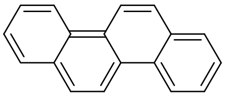
52	Benzo[a]pyrene	50-32-8	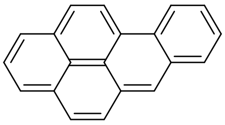
53	Dibenzanthracene	53-70-3	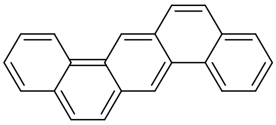
54	Benzo[g,h,i]perylene	191-24-2	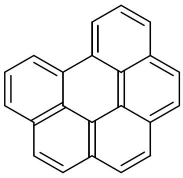
55	Carbamazepine	298-46-4	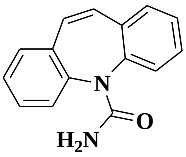
56	17α-Ethinyl estradiol	57-63-6	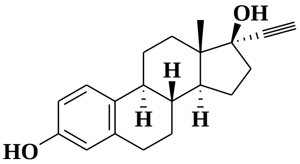
57	3-(4-Methylbenzylidene)camphor	36861-47-9	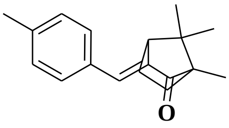
58	Triclosan	3380-34-5	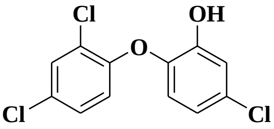

No.	Organic pollutants	CAS	Structures
59	Sulfamethoxazole	723-46-6	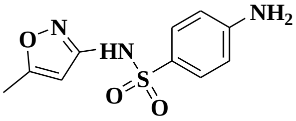
60	Propanolol	525-66-6	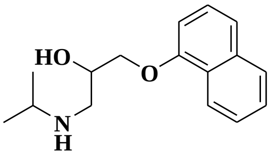
61	Sertraline	79617-96-2	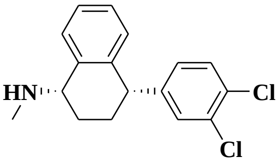
62	p,p'-DDT	50-29-3	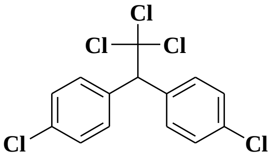
63	o,p'-DDT	789-02-6	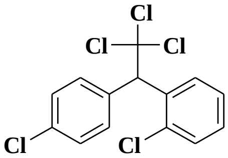
64	p,p'-DDD	72-54-8	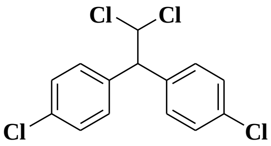
65	o,p'-DDD	53-19-0	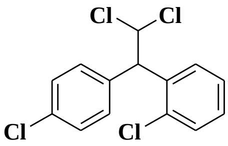
66	p,p'-DDE	72-55-9	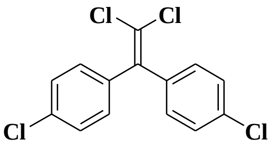
67	o,p'-DDE	3424-82-6	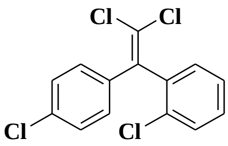
68	p,p'-DDMU	1022-22-6	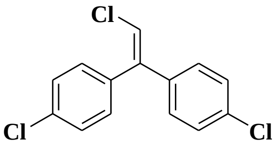
69	4-Fluorobenzoic acid	456-22-4	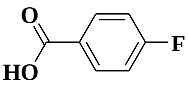

No.	Organic pollutants	CAS	Structures
70	Ethyl benzoate	93-89-0	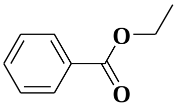
71	Dioctyl phthalate	117-81-7	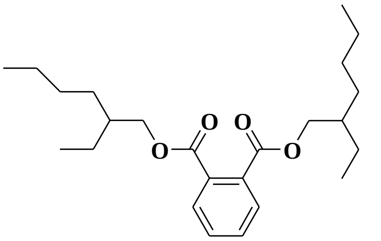
72	Sulfadiazine	68-35-9	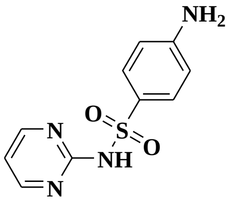
73	Trimethoprim	738-70-5	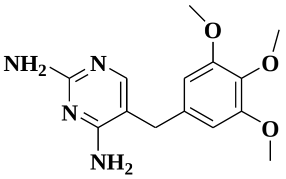
74	Ciprofloxacin	85721-33-1	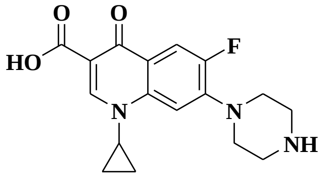
75	Oxytetracycline	79-57-2	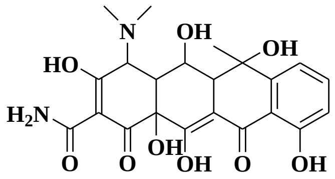
76	Amoxicillin	26787-78-0	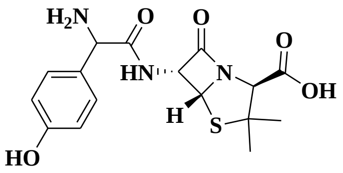
77	Phenprobamate	63-91-2	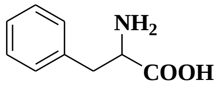
78	Cyclohexane	110-82-7	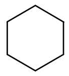
79	α-Hexachlorocyclohexane	319-84-6	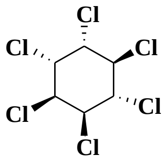

No.	Organic pollutants	CAS	Structures
80	β-Hexachlorocyclohexane	319-85-7	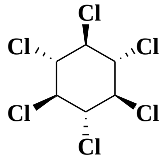
81	γ-Hexachlorocyclohexane	58-89-9	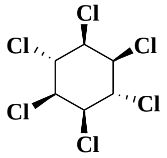
82	δ-Hexachlorocyclohexane	319-86-8	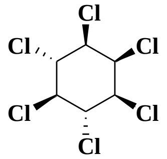
83	Hexane	110-54-3	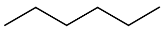
84	Perfluoropentanoic acid	2706-90-3	$F_3C-[CF_2]_3-COOH$
85	Perfluorohexanoic acid	307-24-4	$F_3C-[CF_2]_4-COOH$
86	Perfluoroheptanoic acid	375-85-9	$F_3C-[CF_2]_5-COOH$
87	Pentadecafluorooctanoic acid	335-67-1	$F_3C-[CF_2]_6-COOH$
88	Heptadecafluorooctanesulfonamide	754-91-6	$F_3C-[CF_2]_7-SO_2NH_2$
89	Perfluoro-1-octanesulfonyl fluoride	307-35-7	$F_3C-[CF_2]_7-SFO_2$
90	Perfluorodecanoic acid	335-76-2	$F_3C-[CF_2]_8-COOH$
91	Perfluoroundecanoic acid	2058-94-8	$F_3C-[CF_2]_9-COOH$
92	Perfluorododecanoic acid	307-55-1	$F_3C-[CF_2]_{10}-COOH$

No.	Organic pollutants	CAS	Structures
93	Pentacosafluorotridecanoic acid	72629-94-8	$F_3C-[CF_2]_{11}-COOH$
94	Perfluorotetradecanoic acid	376-06-7	$F_3C-[CF_2]_{12}-COOH$

Table S3 Experimental conditions of log K_d values and size of microplastics

Microplastic	Water type	pH	Temperature range (°C)	Particle size range (μm)	Ref
PE	Seawater	8	18~25	10~440	1~5
PE	Freshwater	7	18~25	10~180	1,5,6
PE	Pure water	7	20~25	20~250	2,7~13
PP	Seawater	8	12~25	75~180,	1
				320~440	3,14
PS	Seawater	8	20~25	3~180	1,15
				320~440	3

Table S4 Values of log D, molecular mass and six quantum chemical descriptors

No	Organic compounds	log D	M_w' (amu.)/100	ε_α a.u.	ε_β a.u.	qH^+ acu	q^- acu	V' (cm^3/mol)/100	π
1	2,3-dichlorobiphenyl	5.050	2.220	0.259	0.321	0.135	−0.122	1.450	1.298
2	2,4'-dichlorobiphenyl	5.050	2.220	0.258	0.317	0.129	−0.135	1.347	1.409
3	2,4,4'-trichlorobiphenyl	5.690	2.560	0.252	0.317	0.147	−0.131	1.675	1.244
4	2,4',5-trichlorobiphenyl	5.690	2.560	0.251	0.321	0.155	−0.125	1.622	1.280
5	2,2',5-trichlorobiphenyl	5.690	2.560	0.263	0.328	0.141	−0.114	1.586	1.258
6	2,2',3,5'-tetrachlorobiphenyl	6.340	2.899	0.259	0.329	0.142	−0.108	1.548	1.392
7	2,2',5,5'-tetrachlorobiphenyl	6.340	2.899	0.257	0.329	0.143	−0.108	1.817	1.190
8	2,4,4',5-tetrachlorobiphenyl	6.340	2.899	0.246	0.321	0.156	−0.125	1.596	1.413
9	2,3',4,4'-tetrachlorobiphenyl	6.340	2.899	0.247	0.322	0.149	−0.130	1.815	1.235

No	Organic compounds	log D	M_w' (amu.)/100	ε_α a.u.	ε_β a.u.	qH^+ acu	q^- acu	V' (cm^3/mol)/100	π
10	3,3',4,4'-tetrachlorobiphenyl	6.340	2.899	0.241	0.318	0.141	−0.109	1.534	1.504
11	2,2',3,5-tetrachlorobiphenyl	6.340	2.899	0.255	0.332	0.156	−0.112	1.850	1.169
12	2,2',4,4'-tetrachlorobiphenyl	6.340	2.899	0.258	0.331	0.149	−0.110	1.716	1.267
13	2,2',3,4',5-pentachlorobiphenyl	6.980	3.239	0.252	0.334	0.157	−0.108	1.827	1.280
14	2,2',3,5',6-pentachlorobiphenyl	6.980	3.239	0.255	0.331	0.149	−0.105	1.920	1.207
15	2,2',4,5',6-pentachlorobiphenyl	6.980	3.239	0.254	0.331	0.157	−0.105	1.833	1.270
16	2,2',4,5,6'-pentachlorobiphenyl	6.980	3.578	0.248	0.336	0.165	−0.103	1.852	1.352
17	2,3,3',4,4'-pentachlorobiphenyl	6.980	3.239	0.243	0.325	0.147	−0.115	1.935	1.239
18	2,3',4,4',5-pentachlorobiphenyl	6.980	3.239	0.242	0.324	0.150	−0.109	1.844	1.311
19	3,3',4,4',5-pentachlorobiphenyl	6.980	3.239	0.235	0.322	0.144	−0.107	1.986	1.246
20	2,2',4,5,5'-pentachlorobiphenyl	6.980	3.239	0.251	0.330	0.159	−0.107	1.667	1.403
21	2,3,3',4',6-pentachlorobiphenyl	6.980	3.239	0.257	0.333	0.149	−0.106	1.828	1.272
22	2,2',3,4',5,6-hexachlorobiphenyl	7.620	3.578	0.248	0.336	0.165	−0.103	1.962	1.276
23	2,2',4,4',5,5'-hexachlorobiphenyl	7.620	3.578	0.248	0.334	0.159	−0.102	1.901	1.324
24	2,3,3',4,4',5-hexachlorobiphenyl	7.620	3.578	0.238	0.329	0.153	−0.107	2.017	1.276
25	3,3',4,4',5,5'-hexachlorobiphenyl	7.620	3.578	0.231	0.327	0.146	−0.097	1.920	1.377
26	2,2',3,4,4',5-hexachlorobiphenyl	7.620	3.578	0.246	0.335	0.154	−0.107	1.733	1.452
27	2,2',3,3',4,5-hexachlorobiphenyl	7.620	3.578	0.247	0.337	0.154	−0.099	2.110	1.084
28	2,2',3,4,4',5'-hexachlorobiphenyl	7.620	3.578	0.246	0.335	0.154	−0.107	2.004	1.256
29	2,2',3,4',5',6-hexachlorobiphenyl	7.620	3.578	0.252	0.335	0.160	−0.101	1.935	1.288
30	2,2',3,3',4,4'-hexachlorobiphenyl	7.620	3.578	0.250	0.338	0.148	−0.095	2.008	1.244
31	2,2',3,3',4,6'-hexachlorobiphenyl	7.620	3.578	0.254	0.335	0.149	−0.101	1.808	1.374
32	2,3,3',4,5,6-hexachlorobiphenyl	7.620	3.578	0.244	0.335	0.135	−0.105	1.812	1.391
33	2,2',3,3',4,4',5-heptachlorobiphenyl	8.270	3.918	0.245	0.338	0.156	−0.093	1.961	1.365
34	2,2',3,4,4',5,5'-heptachlorobiphenyl	8.270	3.918	0.243	0.335	0.160	−0.101	2.132	1.261
35	2,2',3,4',5,5',6-heptachlorobiphenyl	8.270	3.918	0.245	0.336	0.166	−0.097	2.047	1.307
36	Dichlorodiphenyltrichloroethane	5.440	3.519	0.238	0.330	0.132	−0.290	2.089	1.235
37	Chlorobenzene	2.640	1.120	0.282	0.328	0.125	−0.101	0.813	1.068
38	Pentachlorobenzene	5.220	2.479	0.246	0.339	0.168	−0.081	1.340	1.138
39	Hexachlorobenzene	5.860	2.818	0.234	0.344		−0.067	1.415	1.204

No	Organic compounds	log *D*	M_w' (amu.)/100	ε_α a.u.	ε_β a.u.	qH^+ acu	q^- acu	*V*' $(cm^3/mol)/100$	π
40	Benzene	1.990	0.780	0.294	0.330	0.101	−0.101	0.724	0.997
41	Toluene	2.540	0.921	0.294	0.318	0.127	−0.384	0.832	1.062
42	Naphthalene	3.170	1.281	0.255	0.296	0.103	−0.144	1.109	1.241
43	2-Methylanthracene	4.890	1.921	0.233	0.274	0.128	−0.385	1.577	1.534
44	1-methylphenanthrene	4.890	1.921	0.253	0.292	0.130	−0.389	1.579	1.420
45	9,10-Dimethylanthracene	5.440	2.061	0.232	0.268	0.133	−0.388	1.641	1.562
46	3,6-dimethylphenanthrene	5.440	2.061	0.258	0.287	0.129	−0.386	1.660	1.473
47	Phenanthrene	4.350	1.781	0.254	0.294	0.105	−0.157	1.363	1.518
48	Anthracene	4.350	1.781	0.230	0.276	0.105	−0.221	1.370	1.616
49	Pyrene	4.930	2.021	0.236	0.279	0.106	−0.174	1.413	1.794
50	Fluoranthene	4.930	2.021	0.226	0.296	0.111	−0.168	1.565	1.553
51	Chrysene	5.520	2.281	0.243	0.287	0.106	−0.160	1.713	1.661
52	Benzoapyrene	6.110	2.521	0.226	0.271	0.107	−0.253	1.809	1.924
53	Dibenzanthracene	6.700	2.781	0.235	0.283	0.108	−0.238	1.946	1.847
54	Benzo[g,h,i]perylene	6.700	2.301	0.230	0.246	0.135	−0.275	1.825	1.388
55	Carbamazepine	2.250	2.381	0.277	0.311	0.292	−0.636	1.706	1.271
56	17α-Ethinyl estradiol	4.520	2.962	0.293	0.291	0.340	−0.588	2.309	1.169
57	3-(4-Methylbenzylidene)camphor	5.920	2.542	0.231	0.301	0.136	−0.538	2.014	1.352
58	Triclosan	4.660	2.880	0.266	0.311	0.355	−0.571	1.896	1.117
59	Sulfamethoxazole	0.480	2.531	0.263	0.301	0.316	−0.709	1.694	1.163
60	Propanolol	2.600	2.592	0.260	0.284	0.330	−0.565	1.958	1.246
61	Sertraline	5.290	3.051	0.270	0.297	0.247	−0.517	2.413	1.107
62	p,p'-DDT	6.790	3.519	0.241	0.324	0.171	−0.290	2.007	1.284
63	o,p'-DDT	6.790	3.519	0.243	0.325	0.181	−0.307	2.120	1.206
64	p,p'-DDD	5.870	3.180	0.260	0.324	0.230	−0.276	2.087	1.157
65	o,p'-DDD	5.870	3.180	0.259	0.323	0.230	−0.295	1.945	1.235
66	p,p'-DDE	6.000	3.159	0.244	0.311	0.132	−0.282	1.802	1.411
67	o,p'-DDE	6.000	3.159	0.246	0.316	0.132	−0.269	2.176	1.149
68	p,p'-DDMU	5.500	2.820	0.246	0.307	0.171	−0.283	1.716	1.400
69	4-Fluorobenzoic acid	−0.940	1.390	0.292	0.276	0.111	−0.655	0.855	1.112

No	Organic compounds	log *D*	M_w' (amu.)/100	ε_α a.u.	ε_β a.u.	qH^+ acu	q^- acu	*V*' (cm^3/mol)/100	π
70	Ethyl benzoate	2.320	1.501	0.247	0.339	0.126	−0.533	1.242	1.007
71	Dioctyl phthalate	8.390	3.824	0.283	0.305	0.113	−0.331	3.415	1.151
72	Sulfadiazine (pH = 7)	−0.720	2.494	0.267	0.282	0.297	−0.676	1.726	1.187
73	Sulfadiazine (pH = 8)	−1.510	2.491	0.275	0.274	0.285	−0.672	1.739	1.193
74	Trimethoprim (pH = 7)	0.380	2.903	0.273	0.293	0.296	−0.646	2.099	1.160
75	Trimethoprim (pH = 8)	0.730	2.902	0.280	0.291	0.287	−0.650	2.095	1.164
76	Ciprofloxacin (pH = 7)	−1.200	3.311	0.255	0.268	0.377	−0.668	2.259	1.255
77	Amoxicillin (pH = 7)	−2.210	3.646	0.280	0.281	0.364	−0.659	2.532	1.102
78	Oxytetracycline (pH = 8)	−5.590	4.593	0.245	0.258	0.373	−0.663	2.995	1.279
79	Oxytetracycline (pH = 7)	−5.120	4.598	0.231	0.282	0.373	−0.655	2.898	1.292
80	Phenprobamate	−1.280	1.651	0.286	0.321	0.345	-0.605	1.238	1.055
81	Cyclohexane	3.180	0.841	0.385	0.370	0.089	−0.176	0.851	0.943
82	α-Hexachlorocyclohexane	4.260	2.879	0.254	0.386	0.225	−0.043	1.529	1.024
83	β-Hexachlorocyclohexane	4.260	2.879	0.237	0.382	0.216	−0.182	1.460	1.082
84	γ-Hexachlorocyclohexane	4.260	2.879	0.257	0.378	0.225	−0.202	1.471	1.056
85	δ-Hexachlorocyclohexane	4.260	2.879	0.244	0.386	0.223	−0.197	1.559	1.004
86	Hexane	3.290	0.861	0.390	0.382	0.103	−0.324	0.998	0.839
87	Pentadecafluorooctanoic acid	4.000	4.130	0.307	0.300		−0.632	1.548	0.719
88	Perfluoropentanoic acid	1.540	2.630	0.329	0.300		−0.632	1.139	0.628
89	Perfluorohexanoic acid	2.220	3.130	0.321	0.300		−0.633	1.295	0.655
90	Perfluoroheptanoic acid	3.110	3.630	0.312	0.300		−0.632	1.502	0.654
91	Heptadecafluorooctanesulfonamide	5.800	4.990	0.277	0.397	0.341	−0.694	1.801	0.789
92	Perfluoro-1-octanesulfonyl fluoride	6.890	4.999	0.192	0.422	0.411	−0.682	1.931	0.758
93	Perfluorodecanoic acid	5.780	5.130	0.292	0.280		−0.633	2.079	0.663
94	Perfluoroundecanoic acid	6.670	5.630	0.291	0.300		−0.632	2.104	0.715
95	Perfluorododecanoic acid	7.550	6.130	0.289	0.300		−0.632	2.298	0.715
96	Pentacosafluorotridecanoic acid	8.440	6.630	0.285	0.300		−0.632	2.538	0.697
97	Perfluorotetradecanoic acid	9.330	7.129	0.283	0.300		−0.632	2.697	0.704

Table S5 Dissociation information of all ionizable compounds

**Organic pollutants**	**Dissociation sites**	**p*K*a**	**Species distribution**
Sulfadiazine	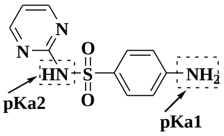	pK_{a1} = 1.57* pK_{a2} = 6.50*	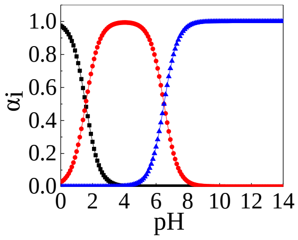
Trimethoprim	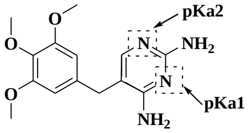	pK_{a1} = 3.23[16] pK_{a2} = 6.76[16]	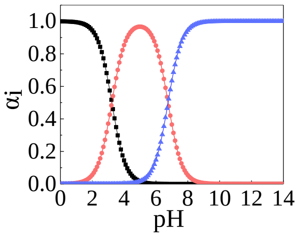
Ciprofloxacin	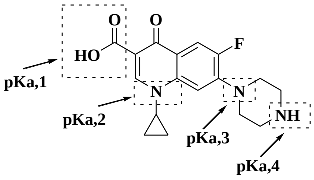	pK_{a1} = 3.01,[16] pK_{a2} = 6.14,[16] pK_{a3} = 8.70,[16] pK_{a4} = 10.58[16]	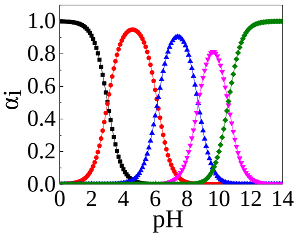
Oxytetracycline	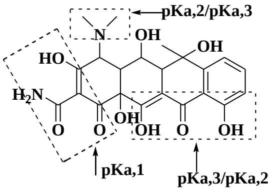	pK_{a1} = 3.22,[16] pK_{a2} = 7.46,[16] pK_{a3} = 8.94[16]	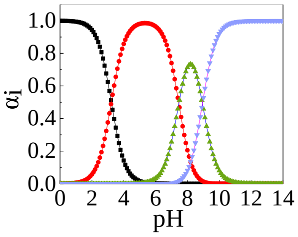
4-Fluorobenzoic acid	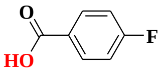	pK_a = 4.14*	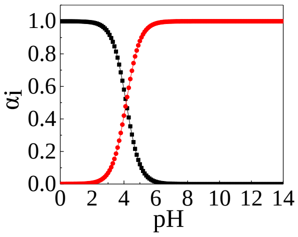
Perfluoropentanoic acid	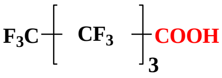	pK_a = 0.54[17]	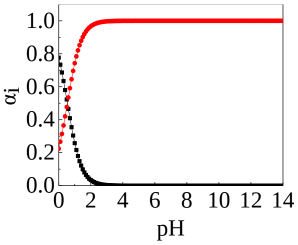

Organic pollutants	Dissociation sites	pK_a	Species distribution
Perfluorohexanoic acid	$F_3C-[CF_3]_4-$**COOH**	pK_a = 0.83[18]	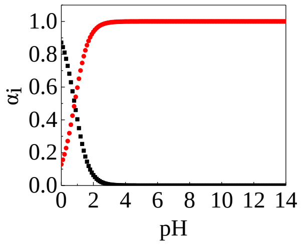
Perfluoroheptanoic acid	$F_3C-[CF_3]_5-$**COOH**	pK_a = 0.47[18]	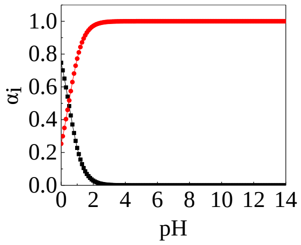
Pentadecafluorooctanoic acid	$F_3C-[CF_3]_6-$**COOH**	pK_a = 0.5[18]	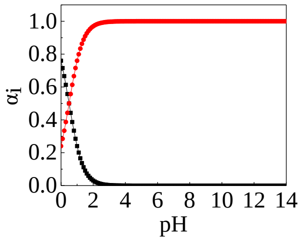
Heptadecafluorooctanesulfonamide	$F_3C-[CF_2]_7-$**SO_2NH_2**	pK_a = 7.01*	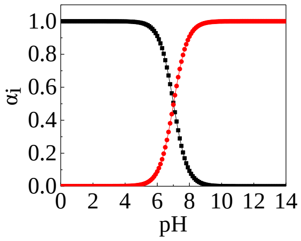
Perfluorodecanoic acid	$F_3C-[CF_3]_8-$**COOH**	pK_a = 2.61[18]	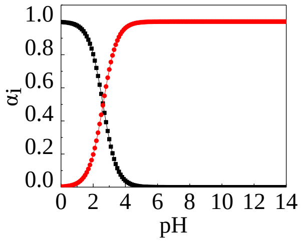
Perfluoroundecanoic acid	$F_3C-[CF_3]_9-$**COOH**	pK_a = 3.13[18]	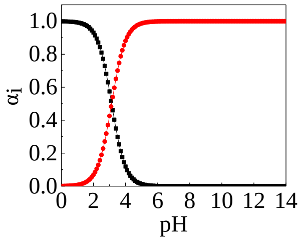
Perfluorododecanoic acid	$F_3C-[CF_3]_{10}-$**COOH**	pK_a = 0.52[18]	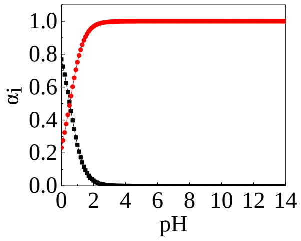

Organic pollutants	Dissociation sites	pK_a	Species distribution
Pentacosafluorotridecanoic acid	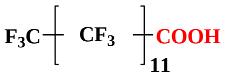	pK_a = 0.52[18]	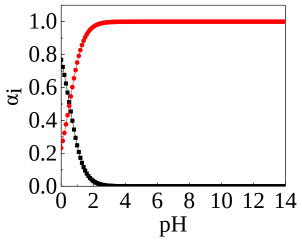
Perfluorotetradecanoic acid	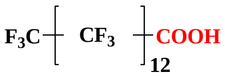	pK_a = 0.37*	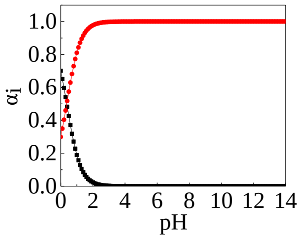

* The p*K*a values were calculate by the software ACD Labs 6.0.[19]

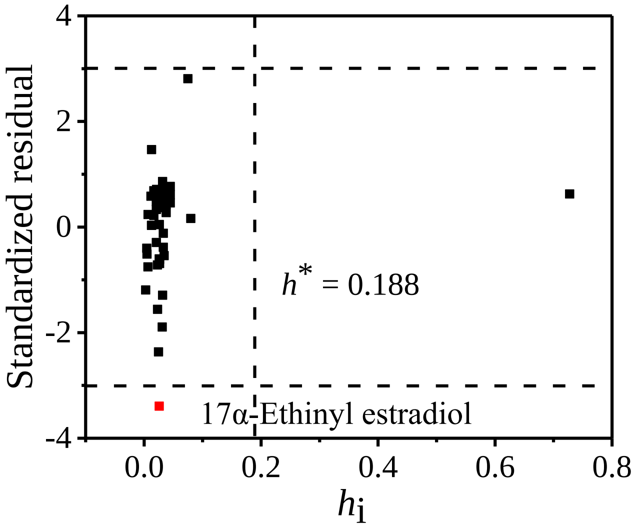

Fig. S1 Williams plots for the applicability domain of model (3). The h_i refers to the verse leverage value.

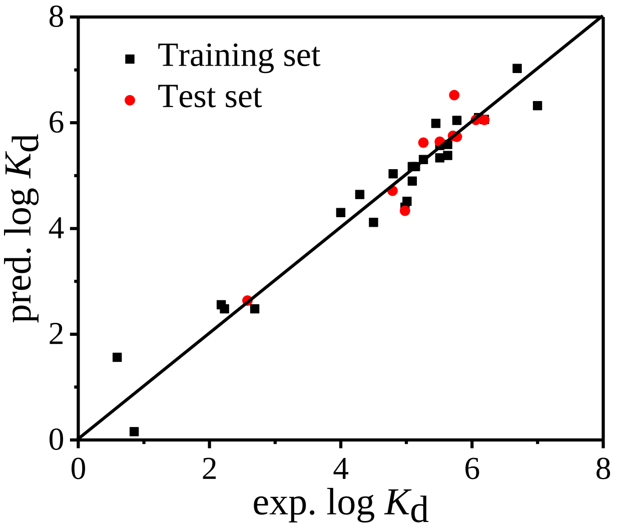

Fig.S2 Fitting plots of experimental and predicted log K_d values by model (5).

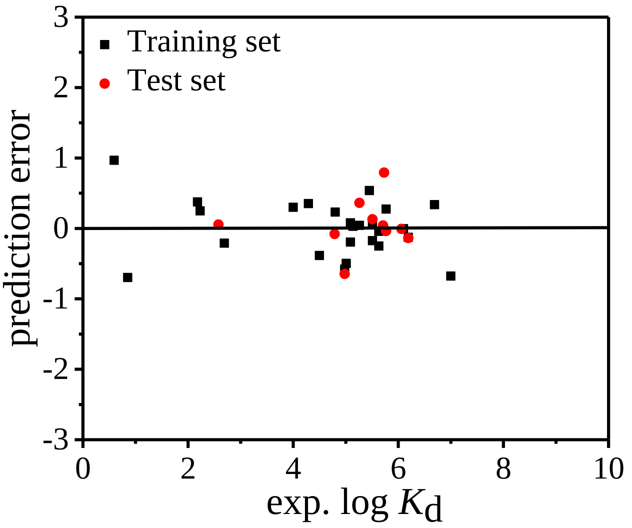

Fig. S3 Distributions of prediction errors of log K_d calculated by model (5).

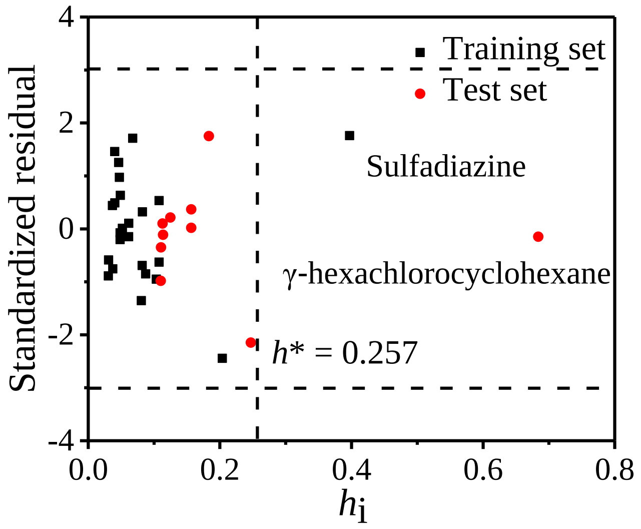

Fig. S4 Williams plot for the applicability domain of model (5). The h_i refers to the verse leverage value.

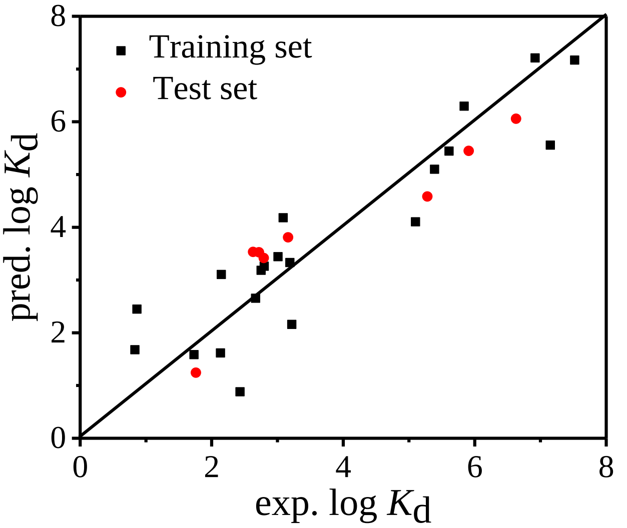

Fig. S5 Fitting plots of experimental and predicted log K_d values by model (6).

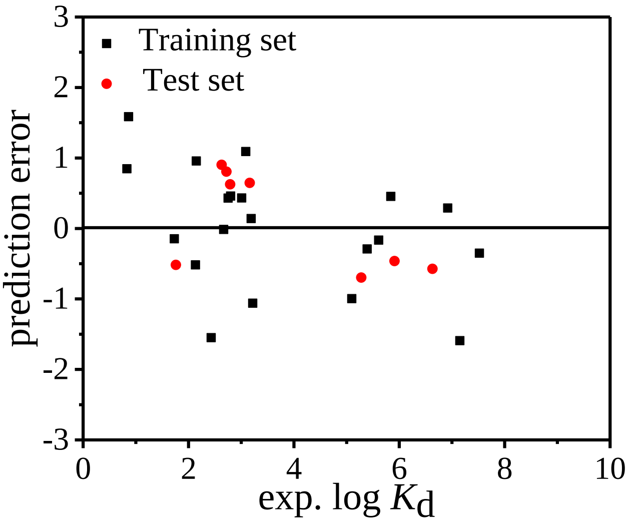

Fig. S6 Distributions of prediction errors of log K_d calculated by model (6).

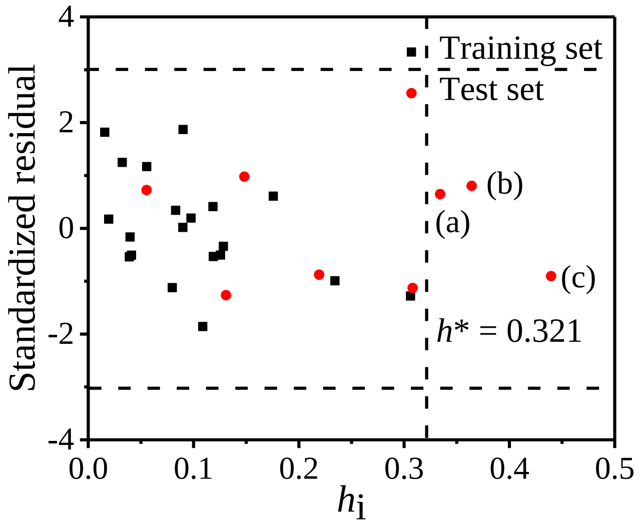

Fig. S7 Williams plot for the applicability domain by model (6). The h_i refers to the verse leverage value. (a): fluoranthene, (b): chrysene, (c): pentacosafluorotridecanoic acid.

References:

1. Li, J., Zhang, K.N., Zhang, H. Adsorption of antibiotics on microplastics. *Environ. Pollut.* **2018**, *237*, 460-467.
2. Zhang, K.N., Li, J., Li, X.Q., Zhang, H. Mechanisms and kinetics of oxytetracycline adsorption-desorption onto microplastics. *Environ. Chem*. **2017**, *36*, 2531-2540.
3. Hwang, L., Won Joon, S., Jung-Hwan, K. Sorption capacity of plastic debris for hydrophobic organic chemicals. *Sci. Total Environ*. **2014**, *470-471*, 1545-1552.
4. Bakir, A., Rowland, S.J., Thompson, R.C. Enhanced desorption of persistent organic pollutants from microplastics under simulated physiological conditions. *Environ. Pollut.*

2014, *185*, 16-23.

5. Velzeboer, I., Kwadijk, C.J.A.F., Koelmans, A.A. Strong sorption of PCBs to nanoplastics, microplastics, carbon nanotubes, and fullerenes. *Environ. Sci. Technol.* **2014**, *48*, 4869-4876.
6. Teuten, E.L., Rowland, S.J., Galloway, T.S. Potential for plastics to transport hydrophobic contaminants. *Environ. Sci. Technol.* **2007**, *41*, 7759-7764.
7. Fernandez, L.A., Macfarlane, J.K., Tcaciuc, A.P. Measurement of freely dissolved PAH concentrations in sediment beds using passive sampling with low-density polyethylene strips. *Environ. Sci. Technol.* **2009**, *43*, 1430-1436.
8. Hüffer, T., Hofmann, T. Sorption of non-polar organic compounds by micro-sized plastic particles in aqueous solution. *Environ. Pollut.* **2016**, *214*, 194-201.
9. Pascall, M.A., Zabik, M.E., Zabik, M.J. Uptake of polychlorinated biphenyls (PCBs) from an aqueous medium by polyethylene, polyvinyl chloride, and polystyrene films. *J. Agr. Food. Chem.* **2005**, *53*, 164-169.
10. Wang, W.F., Wang, J. Different partition of polycyclic aromatic hydrocarbon on environmental particulates in freshwater: microplastics in comparison to natural sediment. *Ecotox. Environ. Safe.* **2018**, *147*, 648-655.
11. Wu, C.X., Zhang, K., Huang, X.L., Liu, J.T. Sorption of pharmaceuticals and personal care products to polyethylene debris. *Environ. Sci. Pollut. R.* **2016**, *23*, 8819-8826.
12. Razanajatovo, R.M., Ding, J.N., Zhang, S.S., Jiang H., Zou, H. Sorption and desorption of selected pharmaceuticals by polyethylene microplastics. *Mar. Pollut. Bull.* **2018**, *136*, 516-523.
13. Hale, S.E., Tomaszewski, J.E., Luthy, R.G., Werner, D. Sorption of dichlorodiphenyltrichloroethane (DDT) and its metabolites by activated carbon in clean water and sediment slurries. *Water Res.* **2009**, *43*, 4336-4346.
14. Mato, Y., Isobe, T., Takada, H., Kanehiro, H., Ohtake, C., Kaminuma, T. Plastic resin pellets as a transport medium for toxic chemicals in the marine environment. *Environ. Sci. Technol.* **2001**, *35*, 318-324.
15. Llorca, M., Schirinzi, G., Martínez, M., Barceló, D., Farré, M. Adsorption of perfluoroalkyl substances on microplastics under environmental conditions. *Environ. Pollut.* **2018**, *235*, 680-691.
16. Qiang, Z.M., Adams, C. Potentiometric determination of acid dissociation constants (p*K*a)

for human and veterinary antibiotics. *Water Res*. **2004**, *38*, 2874-2890.

17. Čabala, R., Nesměrák, K., Vlasáková, T. Dissociation constants of perfluoroalkanoic acids. *Monatsh. Chem*. **2017**, *148*, 1679-1684.
18. Vierke, L., Berger, U., Cousins, I.T. Estimation of the acid dissociation constant of perfluoroalkyl carboxylic acids through an experimental investigation of their water-to-air transport. *Environ. Sci. Technol.* **2013**, *47*, 11032-11039.
19. Advanced Chemistry Development, Inc., Toronto, Ontario, Canada.
